# Folding‐induced Fluorescence Enhancement in a Series of Merocyanine Hetero‐Folda‐Trimers

**DOI:** 10.1002/anie.202114667

**Published:** 2021-11-29

**Authors:** Alexander Schulz, Frank Würthner

**Affiliations:** ^1^ Institut für Organische Chemie and Center for Nanosystems Chemistry Universität Würzburg Am Hubland 97074 Würzburg Germany

**Keywords:** aggregation, dyes/pigments, fluorescence, folding, merocyanines

## Abstract

Many dyes suffer from fast non‐radiative decay pathways, thereby showing only short‐lived excited states and weak photoluminescence. Here we show a pronounced fluorescence enhancement for a weakly fluorescent merocyanine (MC) dye by being co‐facially stacked to other dyes in hetero‐folda‐trimer architectures. By means of fluorescence spectroscopy (lifetime, quantum yield) the fluorescence enhancement was explained by the rigidification of the emitting chromophore in the defined foldamer architecture and the presence of a non‐forbidden lowest exciton state in H‐coupled hetero‐aggregates. This folding‐induced fluorescence enhancement (FIFE) for specific sequences of π‐stacked dyes points at a viable strategy toward improved fluorophores that relates to the approach used by nature in the green fluorescent protein (GFP).

## Graphical abstract

Pronounced fluorescence enhancement could be shown for a weakly fluorescent merocyanine dye in hetero‐folda‐trimer architectures. This folding‐induced fluorescence enhancement (FIFE) for specific sequences of π‐stacked dyes points at a viable strategy towards improved fluorophores that relates to the approach used by nature in the green fluorescent protein (GFP).

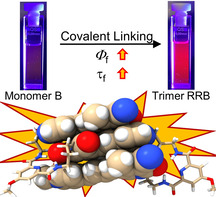

Fluorescence is one of the most useful properties of dyes with widespread applications in biomolecular imaging.[^1^, ^2^, ^3^] Different from absorption properties that are influenced only to a minor extent by the environment,^[4]^ fluorescence is highly sensitive to the fluorophore's surrounding. This is exemplified by common fluorescence quenching pathways such as internal conversion, often associated with conical intersections,[^5^, ^6^] excited state proton or electron transfer[^7^, ^8^] and aggregation‐caused quenching.^[9]^ All of these processes enable fluorescence on/off switching as desired for sensing and imaging applications. Whilst the environmental impact has been considered for many of the most successful fluorophores for the respective applications, for example, DNA intercalation‐induced emission enhancement for thiazole dyes such as TOTO (thiazole orange dimer),^[10]^ there are only few examples where chemists have tried to engineer the dyes’ environment for functional control.^[11]^ Such functional control is accordingly still best illustrated by the natural example of the green fluorescent protein (GFP) from jellyfish.^[12]^ Here an essentially non‐luminescent *p*‐hydroxybenzylidene‐imidazolidinone merocyanine (MC) dye^[13]^ is rigidly embedded within a protein barrel, affording a bright fluorescent dye‐protein construct. Genetic engineering of GFP afforded mutants that cover a large spectral range and were applied widely during the last two decades in biomolecular fluorescence imaging.^[14]^ In contrast, the recovery of the luminescence of this particular chromophore in artificial supramolecular constructs was less successful.^[15]^

MC dyes^[16]^ appear indeed well‐suited to elucidate environmental effects on fluorescence properties. Despite being strong absorbers these dyes exhibit with few exceptions[^17^, ^18^] only very weak fluorescence which has been rationalized by torsional motions leading to conical intersections in the excited state.^[6]^ Accordingly, like in GFP, chromophore embedding within a rigidifying environment may lead to the highly desirable fluorescence enhancement. First evidence for such behavior has been observed in previous research on self‐assembled MC oligomers[^19^, ^20^, ^21^] and on a series of MC dye foldamers bearing up to six identical chromophores of type R (symbol R for the red‐colored solutions of this dye, for definition see Figure 1).[^22^, ^23^]

**Figure 1:**
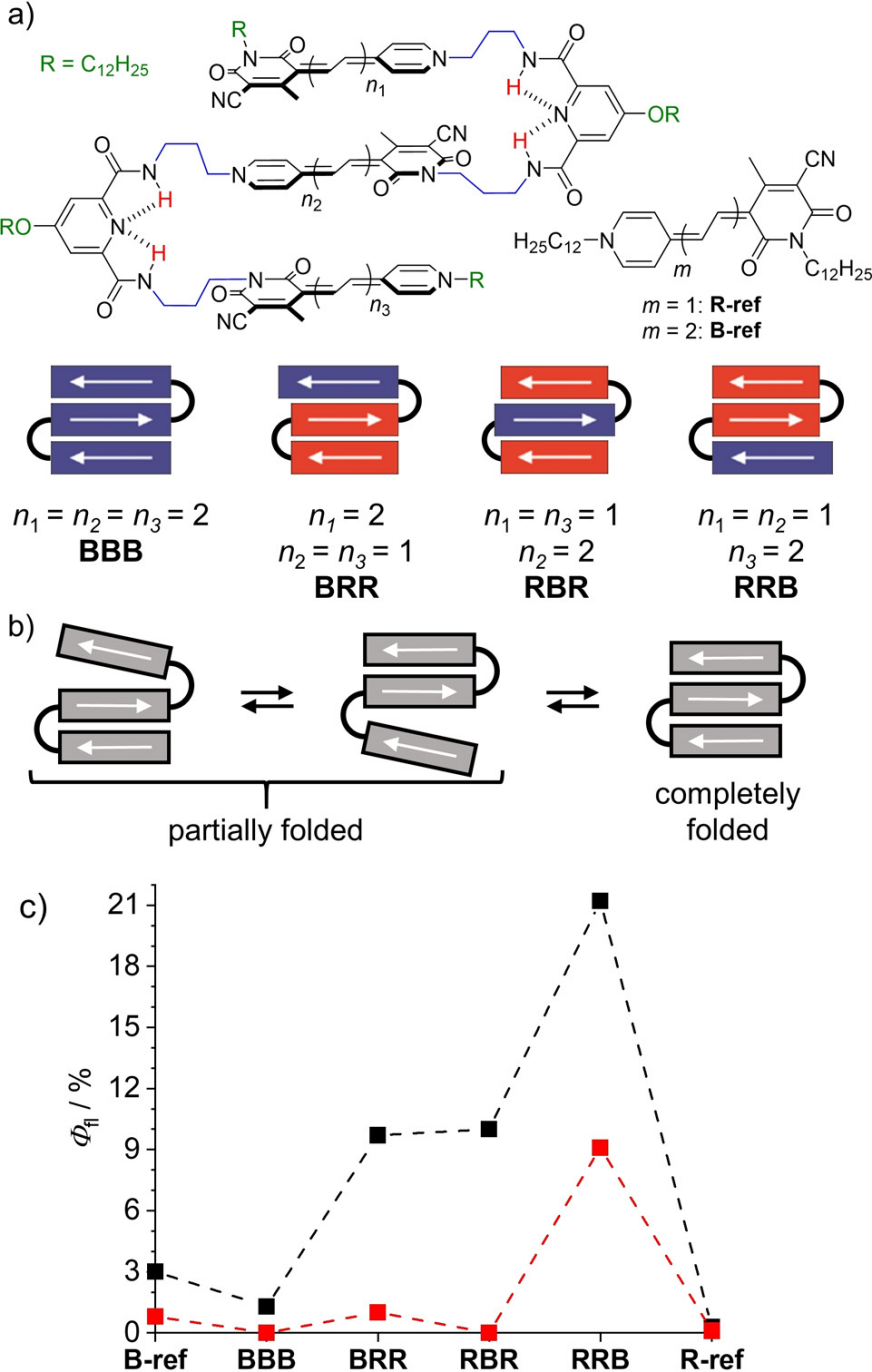
a) Homo‐ and hetero‐folda‐trimers investigated in this study, b) schematic illustration for the solvent‐dependent folding process and c) fluorescence quantum yields determined for the partially folded form in CHCl_3_ (black) and the completely folded stack in CHCl_3_/MCH (red) of the MC trimers at 293 K. Arrows indicate dipole moments in these donor‐acceptor dyes.

However, these dye oligomers suffer strongly from the H‐type exciton coupling in the co‐facially stacked folded state, thereby enabling only modest fluorescence enhancements with fluorescence quantum yields up to 2 % in chloroform/ methylcyclohexane (MCH).^[23]^ Because the best results in our previous work were accomplished for trimers, we herein explore a series of trimers composed of the previously applied dimethine MC dye R (abbreviation related to the red color) and a newly synthesized elongated tetramethine analogue B (abbreviation related to the blue color) (Figure 1 a). Whilst fluorescence properties of such hetero‐foldamers were hitherto never investigated, our previous analyses of exciton coupling in hetero MC aggregates suggested a more beneficial preservation of oscillator strength for the lowest exciton state of these co‐facially stacked dyes, thereby enabling higher radiative rates compared to their homo‐foldamer congeners.[^24^, ^25^]

Figure 1 a shows the molecular structures of the herein investigated folda‐trimers (for synthetic details, see supporting information),^[26]^ Figure 1 b a schematic illustration of their dipole‐dipole interaction^[27]^ driven folding behavior and Figure 1 c our important observation that the hetero‐trimers show significantly increased fluorescence quantum yields (*Φ*_f_) compared to the monomeric reference dyes and the homotrimer **BBB** in the partially folded state. To understand this interesting fluorescence behavior in hetero‐trimers, we first studied the folding behavior of all dyes by NMR and UV/Vis absorption spectroscopy. In general, the folding behavior of all dyes resembles the one of the previously investigated **RRR** homo‐trimer.^[23]^ Thus, the combination of dipole‐dipole interactions between these highly dipolar dyes with the pyridinedicarboxamide turn unit favors the formation of co‐facially stacked dyes with antiparallel oriented dipole moments (Figure 1 b). ^1^H‐NMR spectra for all trimers show only one set of signals in deuterated chloroform and cross‐signals in rotating‐frame Overhauser enhancement spectroscopy (ROESY) support the proposed antiparallel orientation and a close proximity between the adjacent merocyanine dyes (Figure S2–S5). However, NMR cannot distinguish different foldamer species in fast equilibrium, that is, partially and fully folded states that are clearly recognizable in the UV/Vis spectra. As shown in our earlier work for **RRR** the driving force for the formation of π‐stacked dimers in a partially folded structure is indeed much higher than the one for the completely folded state because the third dye is not attracted anymore by strong dipole‐dipole interactions.^[23]^ As a consequence solvents of lower polarity are needed to accomplish a fully folded stack.

Such equilibria between partially folded states and the completely folded trimeric stack as illustrated in Figure 1 b could be also observed in the UV/Vis absorption spectra of the here investigated hetero‐trimers in our standard solvent chloroform (Figure 2 and S6). Among the investigated hetero‐trimers only **RRB** shows a dominant sharp hypsochromically shifted band as expected for a fully folded stack already in chloroform (Figure 2 a, blue dashed line) whilst a decrease of the solvent permittivity by the addition of methylcyclohexane (MCH) is needed to shift the equilibria towards the completely folded state for the other oligomers (Figure 2 a and 2 b, red lines). In the case of the hetero‐trimers **BRR** and **RRB** with the blue dye at an end position this process competes with intermolecular aggregation into extended H‐aggregates as confirmed by additional concentration‐dependent studies (Figure S7). For the latter two hetero‐trimers it accordingly appears that the shorter red chromophore in the middle does not perfectly comply with the geometrical requirements of the turn unit to perfectly accommodate the longer blue dye in a close co‐facial π‐π‐stacking arrangement.

**Figure 2:**
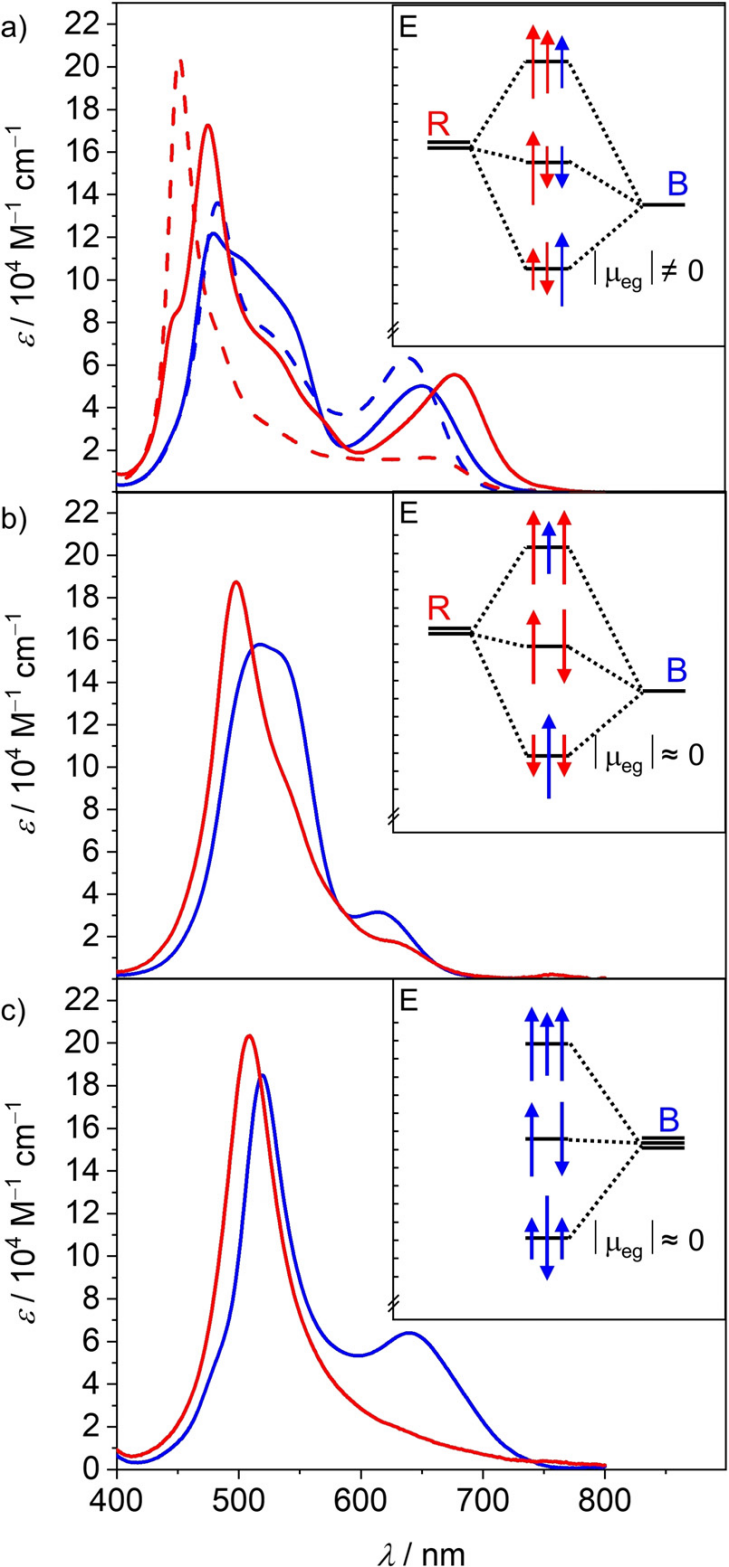
UV/Vis absorption spectra in CHCl_3_ (blue) and CHCl_3_/MCH 1:3 (red) at 293 K for a) **BRR** (solid) and **RRB** (dashed), b) **RBR** and c) **BBB** (*c=*5‐1 μM). The insets show schematic illustrations of the exciton states originating from Coulomb coupling between the neighboring dyes in the fully folded co‐facial stacks and the derived transition dipole moment for the low energy transition.

Owing to their unsymmetrical structure the hetero‐trimers **BRR** and **RRB** retain non‐negligible oscillator strength of the lowest energy transition even in the completely folded stack, leading to the pronounced absorption over 650 nm. In contrast, for **RBR** and **BBB** this transition becomes almost completely forbidden in the completely folded stack, indicating that the absorption around 650 nm in chloroform originates solely from the partially folded structure. This observation can be explained by the interaction of the transition dipole moments according to the molecular exciton theory^[28]^ (Figure 2 insets) and is also nicely reproduced by TDDFT calculations (Figure S10).

Subsequently we studied the emission properties of the trimers in chloroform, in which partially folded and completely folded structures should co‐exist according to the absorption spectra. Indeed, whilst the emission of **BRR** and **RRB** is dominated by a B‐monomer‐like band at around 690 nm, an additional broad and red‐shifted emission above 750 nm is visible upon excitation at the sharp, high energy band originating from the completely folded stack (Figure 3 a and Figure S8 b). We therefore attribute the sharp emission to the partially folded state and the exciplex‐like broader emission to the completely folded stack. In case of the trimers **RBR** and **BBB**, for which the transition to the lowest excited state becomes forbidden for the fully stacked ensemble, only the monomer‐like emission can be observed regardless of the excitation wavelength (Figure 3 b and Figure S7 a), indicating that the fluorescence of the completely folded stack is quenched due to the forbidden nature of the transition from the lowest exciton state to the ground state (Figure 2 b,c). This further suggests that the folding‐unfolding process is much slower than the deactivation of the excited states. We adjusted our determination method of *Φ*_f_ (Figure S1 and Table S1) and the fluorescence lifetime (*τ*_f_; see Table S2) to accommodate for this behavior. The obtained *Φ*_f_ for the partially folded structure shows that while **BBB** (1.3 %) is quenched in comparison to **B‐ref** (3 %), the hetero‐trimers **BRR**, **RBR** and **RRB** all show pronounced folding‐induced fluorescence enhancement (FIFE), resulting in a quantum yield of up to 21 % for **RRB** and 10 % for the two other trimers (Figure 1 c).

**Figure 3:**
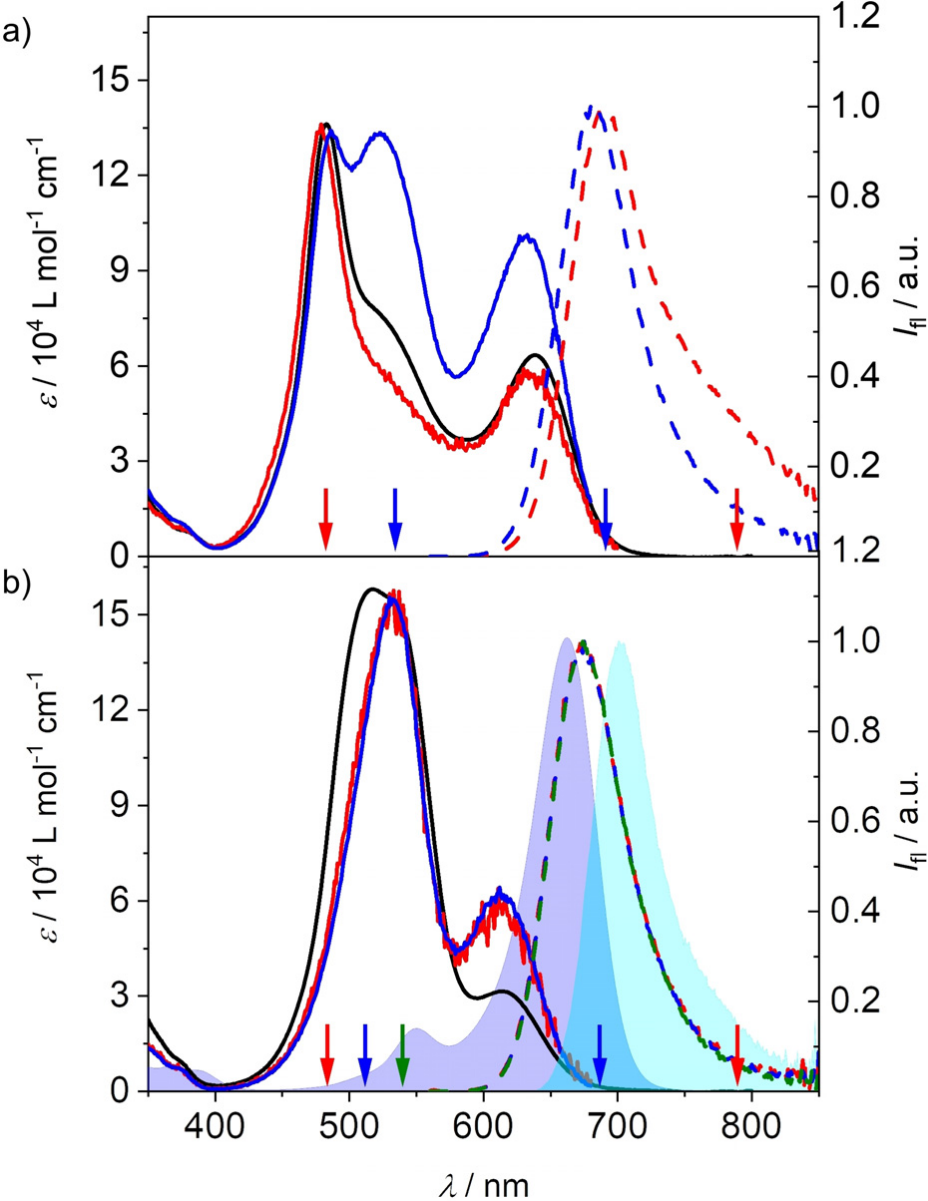
Absorption (black), excitation (red, blue; solid) and emission (colored; dashed) spectra of a) **RRB** and b) **RBR** in CHCl_3_ at 293 K. The excitation and emission wavelengths are indicated by arrows in the respective color. The absorption (dark blue area) and emission (light blue area) spectra of **B‐ref** are included in b) for reference.

To gain further insight into the photophysical processes in the excited state we carried out time‐dependent fluorescence measurements (Figure 4 a). For the folda‐trimers *τ*_f_‐values of 0.6 (**BBB**) to 1.5 ns (**RBR**) were determined in chloroform, which are significantly longer compared to the ones of the monomeric references (ca. 0.1 ns). From these values the radiative (*k*_r_) and non‐radiative (*k*_nr_) rate constants for all trimers were calculated (Figure 4 b and Table S3).

**Figure 4:**
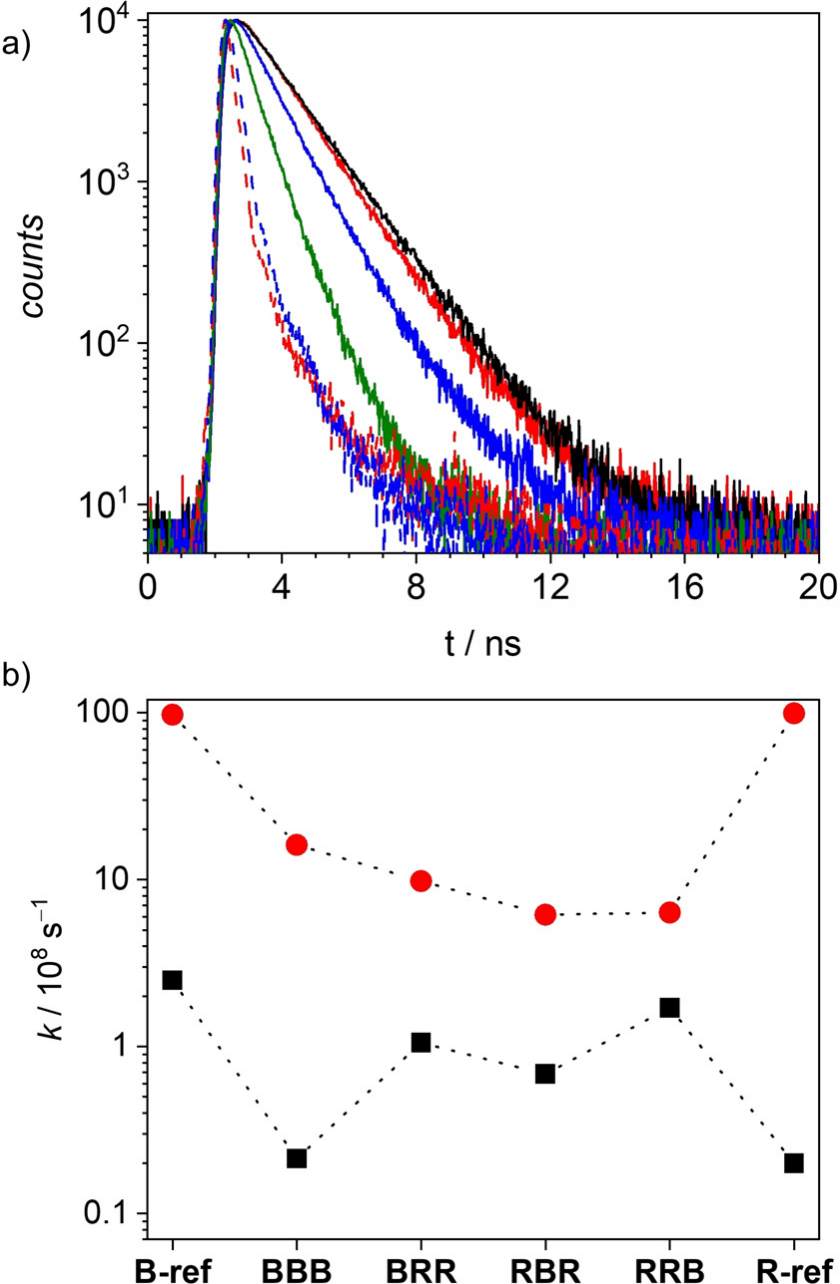
a) Fluorescence decays for **RBR** (black), **RRB** (red), **BRR** (blue), **BBB** (green), **B‐ref** (blue, dashed) and **R‐ref** (red, dashed) in chloroform at 293 K and (b) the radiative (black) and non‐radiative (red) rate constants calculated from *τ*_f_ and *Φ*_f_.

As expected for co‐facially stacked structures with H‐type coupling, the forbidden nature of the radiative transition from the lowest exciton state leads to a reduction of *k*_r_ compared to the monomeric references. This effect is most pronounced for the homo‐trimer **BBB**, while the expected preservation of *k*_r_ could be observed for the hetero‐trimers in accordance with the model shown in the insets of Figure 2. Furthermore, a reduction of *k*_nr_ can be observed for all trimers, which we attribute to the rigidification of the emitting type B chromophore by the adjacent chromophores, hindering the common deactivation pathway for merocyanines via a conical intersection.[^5^, ^6^] This rigidification seems to be least effective in the case of **BBB**, which we attribute to the existence of one weakly associated B chromophore in the partially folded structures, while for the hetero‐trimers a more effective rigidification can be observed, indicating close proximity of the B chromophore to at least one adjacent R chromophore even in the partially folded structure. In the symmetrical trimer **RBR** both rates show the strongest reduction, which can be rationalized by the position of the B chromophore in the center of the structure leading to an efficient rigidification but also a more forbidden lowest exciton H‐state (Figure 2 b, inset). **RRB** shows a similar rigidification but more beneficial preservation of *k*_r_ leading to the increased *Φ*_f_. Surprisingly, the other unsymmetrical congener **BRR** shows a lower *k*_r_ and higher *k*_nr_ compared to **RRB**, indicating less effective rigidification and stronger H‐coupling.

To investigate the reason for this unexpected result we measured excitation spectra for all trimers at their monomer‐like emission band at 690 nm (Figure 5 a). The different intensity and spectral location of the maxima observed for the long wavelength band in the excitation spectra (blue shaded area) indicate a different environment of the B chromophore in all three hetero‐trimers. Notably, the trends observed for the shift and relative intensity coincide with the trends observed for *k*_nr_ and *k*_r_, respectively. This suggests that the observed shift is primarily attributable to a different polarization of the chromophore by the environment in the foldamer structure, while the decrease in intensity is attributable to H‐type coupling as illustrated in the insets of Figure 2.^[29]^ The different environmental influence on the blue dye at the end position of the unsymmetrical trimers can also be observed in the DFT optimized structure, which shows a favorable non‐covalent contact between the B chromophore and the adjacent turn unit in **RRB** providing additional rigidification (Figure 5 b). This interaction is absent in **BRR** which is in agreement with the higher *k*_nr_‐value for this trimer compared to **RRB**. Further, the calculated exciton coupling *J* between adjacent chromophores^[30]^ in the BR substructure is significantly higher in **BRR** (2363 cm^−1^) compared to **RRB** (1629 cm^−1^) resulting in a stronger contribution of the center R dye to the lowest excited state and therefore a reduction of the transition dipole moment in **BRR** (Figure 2 inset), which is in good agreement with the obtained *k*_r_ (Table S5).

**Figure 5:**
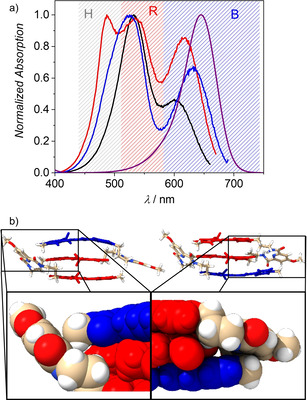
a) Normalized excitation spectra of **RBR** (black), **RRB** (red), **BRR** (blue) and **B‐ref** (purple) in chloroform at 293 K (*λ*_em_=690 nm) and b) the geometry optimized structures (B97D3/def2SVP) of **BRR** (left) and **RRB** (right).

In accordance to the measurements in chloroform, only **RRB** and **BRR** show notable emission from the completely folded stack in CHCl_3_/MCH 1:3 (Figure 1 c and S9). While for both trimers a superposition of the monomer‐like emission (vide supra) and the exciplex‐like emission could be observed, isolated emission from the completely folded stack could only be obtained for **RRB** upon excitation at higher energies. The acquired emission spectrum was found to be rather broad with a red‐shifted maximum of 750 nm (Figure S10). While the *Φ*_f_ of **B‐ref** was almost entirely quenched in this solvent system due to the formation of self‐assembled dimers,^[27]^ the *Φ*_f_ of the exciplex‐like emission of **RRB** was determined to be around 10 % (Table S4). The lifetime of the latter was furthermore found to be significantly increased compared to the partially folded state. The derived rate constants *k*_nr_ and *k*_r_ for this fully folded structure are indeed further reduced compared to the partially folded one which can be rationalized by a reinforced rigidification in the completely folded stack and a more pronounced H‐coupling, respectively. In this case the reduction in *k*_r_ due to the more forbidden character of the low energy transition is more pronounced than the additional rigidification, thereby leading to the reduction of *Φ*_f_ (Table S4). By using the more viscous liquid paraffin solvent to create a more rigid environment, the *Φ*_f_ of the exciplex‐like emission of **RRB** could be further enhanced to about 14 % compared to 2 % for **B‐ref** in the same solvent mixture.

In conclusion, the absorption and emission properties of a series of foldamers composed of three merocyanine dyes have been investigated. We were able to discern the beneficial and detrimental effects on the emission of these typically only weakly luminescent chromophores upon organization in β‐sheet‐like foldamer structures. We found that rigidification and H‐coupling strongly depend on the position of the dye with the smallest band gap (most bathochromic absorption) in the stack and its surrounding environment. Whilst the used monomeric dyes had fluorescence quantum yields of <3 % that were even reduced in their homo‐trimers, the best hetero‐trimer ensemble showed a significant folding‐induced fluorescence enhancement with a fluorescence quantum yield of up to 21 % in the far‐red to NIR spectral region. We envision that this approach of chromophore rigidification by an embedding environment can be applied to a large variety of dyes to afford GFP‐like fluorescence enhancements.

## Conflict of interest

The authors declare no conflict of interest.

## Supporting information

As a service to our authors and readers, this journal provides supporting information supplied by the authors. Such materials are peer reviewed and may be re‐organized for online delivery, but are not copy‐edited or typeset. Technical support issues arising from supporting information (other than missing files) should be addressed to the authors.

Supporting Information
