# Supplementary material for: Folding‐induced Fluorescence Enhancement in a Series of Merocyanine Hetero‐Folda‐Trimers
Source: Angew Chem Int Ed Engl. 2021 Nov 29;61(2):e202114667. doi: 10.1002/anie.202114667 (PMC9299730; doi:10.1002/anie.202114667)
Supplement: Supplementary file 1 — Supporting Information [file ANIE-61-0-s001.pdf]

Angewandte Chemie
Eine Zeitschrift der Gesellschaft Deutscher Chemiker

Supporting Information

Folding-induced Fluorescence Enhancement in a Series of Merocyanine Hetero-Folda-Trimers

*Alexander Schulz and Frank Würthner**

anie_202114667_sm_miscellaneous_information.pdf

Table of Contents

Experimental
Procedures S2
Synthesis and characterization S4
2D NMR studies S13
UV-vis and fluorescence studies.................................... S17
TD-DFT calculations..S20
NMR spectra.. S21
Mass spectra...S31
Additional references.. S34

Experimental Procedures

All commercially available starting materials and reagents were used without further purification. Anhydrous dichloromethane was dispensed from a solvent purification system (Innovative Technology). Organic solvents for spectroscopic studies were of spectroscopic grade and used without further purification. Reference **R-ref** and building blocks **Ra-c** were synthesized according to previously reported procedures.[S1,S2] Analytical thin layer chromatography (TLC) was performed on silica gel plates (Merck 60F254) and visualized with a UV lamp (254 nm). Column chromatography was performed with commercial glass columns using silica gel 60M (particle size 0.04-0.063 mm).
Melting points: were determined with a BÜCHI Melting Point B-545 apparatus and are uncorrected.

UV/Vis spectroscopy: UV/Vis absorption spectra were recorded on a JASCO V670 or V770 spectrometer with a scan rate of 100 nm/min and a data interval of 0.5 nm. Conventional quartz cells from 1 mm to 100 mm path length were used to cover different concentrations throughout the study. Organic solvents for spectroscopic studies were of spectroscopic grade and used without further purification.

Mass spectrometry: High-resolution electrospray ionization time-of-flight (HRESI-TOF) mass spectra were measured in the positive ion mode on a Bruker Daltonic microTOF-Q III spectrometer.

NMR spectroscopy: NMR spectra (1H, 13C, 1H-1H COSY, 1H-1H ROESY) were recorded on a Bruker Avance III HD 400 spectrometer in $CDCl_3$ at 295 K. The residual solvent signals were used as internal standard ($CDCl_3$: ^1H: δ = 7.26 ppm, ^{13}C: δ = 77.16 ppm; CD_2Cl_2: ^1H: δ = 5.32 ppm, ^{13}C: δ = 53.84 ppm)[S3] and the chemical shifts δ are reported in ppm. Abbreviations used for signal multiplicity are: s = singlet, d = doublet, t = triplet, q = quartet, m = multiplet or overlap of nonequivalent resonances, br = broad. Coupling constants, *J*, are reported in Hertz (Hz). Data processing was performed with the Topspin software.
Computational details: For the simulation of UV/vis spectra, all structures were optimized in the frame of density functional theory (DFT) using the B3LYP[S4] functional together with the def2-SVP[S5] basis set in Gaussian09.[S6] The 10 lowest excited states were calculated for every molecule using time-dependent density functional theory (TD-DFT) in Gaussian09. Correct long-range behavior of the method was ensured by using the long-range corrected hybrid density functional ωb97 together with the same basis set as described above.[S7] Broadened spectra were obtained by convolution with Gaussians of 0.2 eV width. In order to compensate the overestimation of transition energies by the employed functional, a shift to lower energies was applied to all calculated TD-DFT spectra. In all DFT calculations, Grimme's empirical D3 correction was used to account for the correct dispersion interactions.[S8]

Fluorescence spectroscopy and quantum yield determination: Steady-state absorption spectra were recorded using a V770 UV-Vis spectrometer (JASCO Inc., Japan). Emission and excitation spectra were measured with a FLS980-D2D2-ST (Edinburgh Instruments Ltd., UK) fluorescence spectrometer and corrected against the photomultiplier sensitivity and the lamp intensity. All spectra were recorded at 293 K, if not stated otherwise, and the temperature was controlled by a sample holder with Peltier element. The fluorescence quantum yields (Φ_{fl}) were determined as average value of four different excitation wavelengths relative using *N*,*N*'-bis(2,6-diisopropylphenyl)-1,6,7,12-tetraphenoxy-perylene-3,4:9,10-bis(dicarboximide) (Φ_{fl} = 0.96 in chloroform)[S9] and Rhodamine 800 (Φ_{fl} = 0.25 in ethanol)[S10] as standards under highly diluted conditions (OD ≤ 0.05) and magic angle conditions (54.7°). The fluorescence lifetimes were determined by Time Correlated Single Photon Counting (TCSPC) using a EPL510 pulsed diode laser (λ_{exc} = 505.8 nm) with a pulse width of 141.7 ps or a EPL485 pulsed laser diode (λ_{exc} = 479.7 nm) with a pulse width of 113.8 ps with an FLS980-D2D2-ST spectrometer (Edinburgh Instruments Ltd., UK) under magic angle conditions (54.7°). The fitting of the data was carried out using the Recursive-Fit-routine of the FAST software supplied by Edinburgh Instruments Ltd., Inc. which corrects against the instrument response function (IRF).
The existence of an ensemble of inhomogeneously folded states with presumably different emission properties means the determined Φ_{rel} can be described as the sum of the different quantum yields times the ratio of species excited X:

$$\Phi_{\mathrm{rel}} = \sum \Phi_{\mathrm{i}} * X_{\mathrm{i}} = \Phi_{\mathrm{open}} * X_{\mathrm{open}} + \Phi_{\mathrm{folded}} * X_{\mathrm{folded}} + \underbrace{\sum \Phi_{\mathrm{n}} * X_{\mathrm{n}}}_{\text{other species}} \quad (1)$$

As we only observed emission which we attribute to a partially folded (open) or a completely folded form we assume any other possible species to be negligible. Therefore, the term can be simplified to:

$$\Phi_{\mathrm{rel}} = \sum \Phi_{\mathrm{i}} * X_{\mathrm{i}} \approx \Phi_{\mathrm{open}} * X_{\mathrm{open}} + \Phi_{\mathrm{folded}} * X_{\mathrm{folded}} \quad (2)$$

To minimize contamination of the spectrum with the other emissive species, we measured an emission map over a wide range of excitation wavelengths. On the next slide our approach is explained in detail for the partially folded form of **RRB** in $CHCl_3$, but the same approach was used for the completely folded species of **RRB** in $CHCl_3$/MCH 25:75 and for the partially folded species of the other trimers.

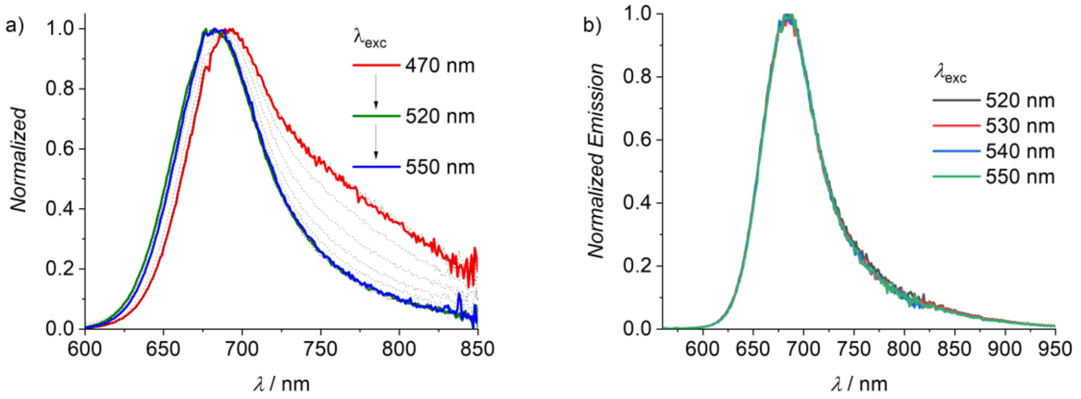

Figure S1. Emission spectra of **RRB** at different excitation wavelengths using a) a visible PMT and b) a NIR PMT in $CHCl_3$ at 293 K ($c \approx 0.1$ µM).

From these spectra we can see that the emission spectra at excitation wavelengths over 520 nm are almost identical, indicating that the amount of excited completely folded species is negligible at these wavelengths. Therefore equation 2 can be further simplified.

$$\Phi_{\mathrm{rel}} \approx \Phi_{\mathrm{open}} * X_{\mathrm{open}} \tag{3}$$

Table S1. Relative fluorescence quantum yield determined for the partially folded species of **RRB** at different excitation wavelengths in $CHCl_3$ at 293 K (c ≈ 0.1 µM).

λ_{exc} / nm[a]	530	535	540	545	550
Φ_{rel}	21.2%	21.5%	21.4%	21.4%	20.7%

[a] For the determination of the quantum yield a slit width of 3.7 nm for excitation and emission monochromator path was chosen.

From this we conclude a quantum yield of about 21.4% for the partially folded form of **RRB**. For the lifetime determination we used a variation of the excitation and detection wavelengths to assign the different contributions to one species.

Table S2. Fluorescence lifetime determined for **RRB** with different pulsed laser diodes and detection wavelengths in $CHCl_3$ at 293 K (c ≈ 0.1 µM).

	λ_{em} = 690 nm	740 nm	790 nm[a]
EPL485 λ_{exc} = 480 nm	τ_1 = 1.06 ± 0.01 ns (69%) τ_2 = 2.01 ± 0.002 ns (31%) χ^2 = 1.002	τ_1 = 1.16 ± 0.04 ns (48%) τ_2 = 2.19 ± 0.01 ns (52%) χ^2 = 1.170	τ_1 = 1.1 fixed (25%) τ_2 = 2.18 ± 0.0022 ns (75%) χ^2 = 1.325
EPL510 λ_{exc} = 506 nm	τ_1 = 1.24 ± 0.009 ns (87%) τ_2 = 1.99 ± 0.02 ns (13%) χ^2 = 1.118		

[a] For this emission wavelength only small intensities could be detected even with large slit widths, leading to a rather poor signal to noise ratio. Without fixation the fit gave different values for both lifetimes with a similar χ^2 value.

From this we can assign the short lifetime of about 1.1-1.2 ns to the partially folded form, which has its emission maximum at 690 nm and the longer lifetime of about 2.0-2.2 ns to the completely folded stack, which has its emission maximum at around 790 nm.

Results and Discussion

Synthesis and characterization

The syntheses of precursors **1a**, **1b**, **2a**, **2b**,building blocks **Ra-c** and dimer **RR-OH** are described in the literature.[1],[2]

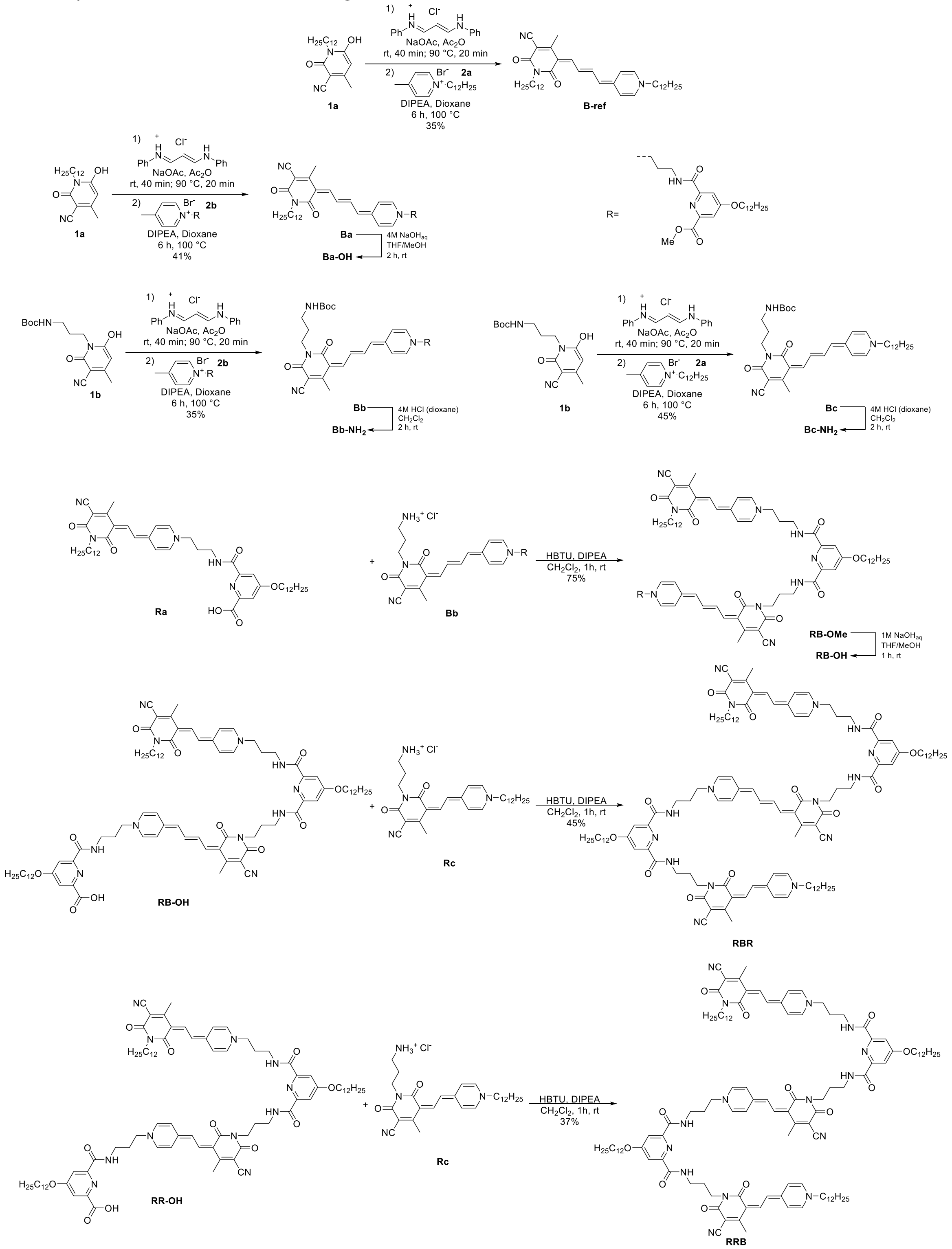

Scheme S1. Synthesis of merocyanine building blocks **Ba-c**, trimers **RBR** and **RRB**. HBTU = (2-(*1H*-benzotriazol-1-yl)-1,1,3,3-tetramethyluronium hexafluorophosphate, DIPEA = *N,N*-diisopropylethylamine.

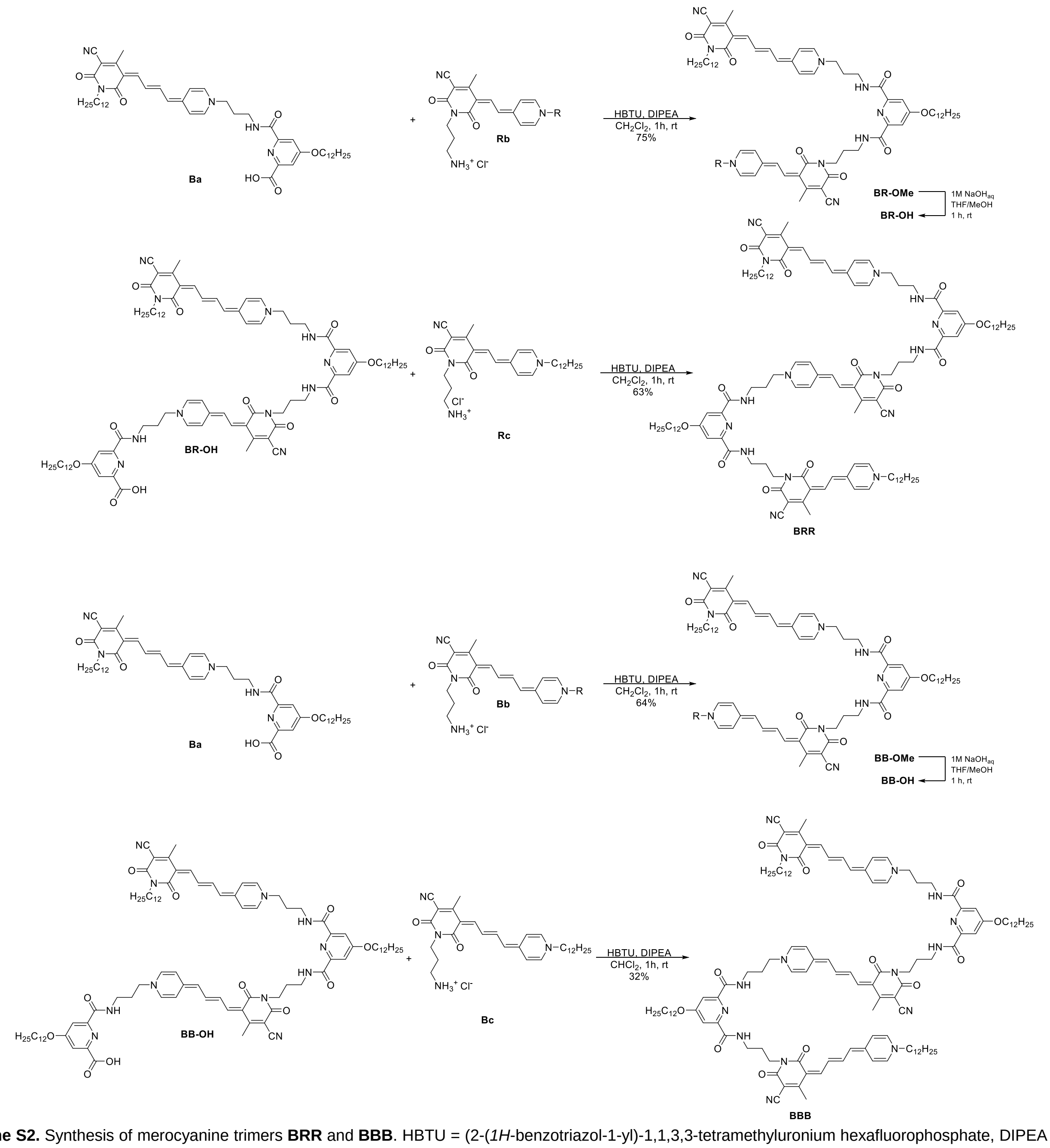

Scheme S2. Synthesis of merocyanine trimers **BRR** and **BBB**. HBTU = (2-(*1H*-benzotriazol-1-yl)-1,1,3,3-tetramethyluronium hexafluorophosphate, DIPEA = *N,N*-diisopropylethylamine.

*Synthesis of **B-ref***

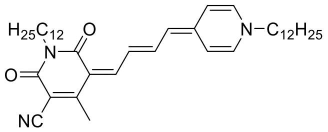

Malonaldehyde dianilide hydrochloride (200 mg, 773 µmol, 1.5 equiv.), hydroxypyridone **1a** (246 mg, 773 µmol,1.3 equiv.) and sodium acetate (63.4 mg, 773 µmol, 1.5 eq) were suspended in 2 mL acetic anhydride. The suspension was stirred at room temperature for 20 min, followed by 20 min at 90 °C. After cooling down to room temperature, the solution was dilluted with diethylether (100 mL) and cooled to −20 °C overnight. The formed precipitate was filtered off and directly mixed with 1-dodecyl-4-methylpyridin-1-ium bromide (**2a**) (204 mg, 595 µmol, 1 equiv.) in 30 mL 1,4-dioxane containing 104 µL of Hünig's base (595 µmol, 1 equiv.). The mixture was heated to 100 °C and stirred at this temperature for 6 h. The reaction mixture was allowed to cool to room temperature and the solvent was evaporated under vacuum. The obtained crude product was purified by column chromatography (eluent: dichloromethane/MeOH = 96/4,v/v%), size-exclusion chromatography (SX1, CH_2Cl_2/MeOH = 9:1) and precipitation from MeOH to yield compound **B-ref** as a purple solid (128 mg, 35%). **Mp.**: 235–238 °C (from CH_2Cl_2). **^{1}H NMR** (400 MHz, CD_2Cl_2): δ (ppm) = 7.82 (dd, J_1 = 13.6 Hz, J_2 = 11.8 Hz, 1H), 7.68 (d, J = 7.1 Hz, 2H), 7.48 (dd, J_1 = 14.3 Hz, J_2 = 11.8 Hz, 1H), 7.27 (d, J = 7.1 Hz, 2H), 7.12 (d, J = 13.6 Hz), 6.24 (d, J = 14.3 Hz), 4.11 (t, J = 7.4 Hz, 2H), 3.90 (t, J = 7.5 Hz, 2H), 2.30 (s, 3H), 1.91-1.84 (m, 2H), 1.60-1.52 (m, 2H), 1.37-1.22 (m, 36H), 0.88 (m, 6H). **^{13}C NMR** (101 MHz, CD_2Cl_2): δ (ppm) = 163.6, 163.4 154.8, 154.4, 151.3, 145.3, 140.4, 121.9, 120.5, 120.1, 116.6, 107.5, 87.6, 60.1, 39.9, 32.3, 32.29, 31.3, 30.1, 30.0, 29.97, 29.8, 29.77, 29.71, 29.3, 28.6, 27.8, 26.5, 23.1, 23.08, 18.8, 14.29, 14.27; **HRMS** (ESI, positive mode): *m/z* calcd for $C_{40}H_{62}N_3O_2$ [M + H]$^+$: 616.4842, found: 616.4872; UV/Vis ($CHCl_3$): λ_{max} = 663 nm (ε = 163000 $M^{-1}cm^{-1}$).

General Procedure for the synthesis of type B building blocks (Ba-c)

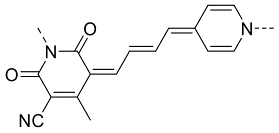

Malonaldehyde dianilide hydrochloride (1.5 equiv.), the respective hydroxypyridone **1a** or **1b** (1.3 equiv.) and sodium acetate (1.5 eq) were suspended in acetic anhydride. The suspension was stirred at room temperature for 20 min, followed by 20 min at 90 °C. After cooling down to room temperature, the solution was dilluted with diethylether and cooled to −20 °C overnight. The formed precipitate was filtered off and the obtained solid directly mixed with the respective pyridine moiety **2a** or **2b** (1 equiv.) in 1,4-dioxane containing Hünig's base (1 equiv.). The mixture was heated to 100 °C and stirred at this temperature for 6 h. The reaction mixture was allowed to cool down to room temperature and then solvent was evaporated under vacuum. The obtained crude product was purified by column chromatography (eluent: CH_2Cl_2/MeOH = 96/4 v/v% for **Ba** and **Bc**; 94/6 v/v% for **Bb**) and size-exclusion chromatography (SX1, CH_2Cl_2/MeOH = 9:1) to yield the desired compound.

*Building block **Ba***

Dark blue solid (151 mg, 41%). **Mp.**: 101–104 °C (from CH_2Cl_2). **^{1}H NMR** (400 MHz, CD_2Cl_2): δ (ppm) = 8.45 (t, J = 6.8 Hz,), 8.03 (d, J = 7.5 Hz, 2H), 7.78-7.72 (m, 2H), 7.54 (d, J = 2.7 Hz, 1H), 7.22-7.16 (m, 3H), 6.85 (d, J = 14.6 Hz), 6.13 (d, J = 14.6 Hz), 4.35 (t, J = 6.3 Hz, 2H), 4.06 (t, J = 6.6 Hz, 2H), 3.93 (s, 3H), 3.91-3.85 (m, 2H), 3.54-3.51 (m, 2H), 2.28-2.25 (m, 2H), 2.14 (s, 3H), 2.00-1.96 (m, 2H), 1.80-1.73 (m, 4H), 1.53-1.49 (m, 4H), 1.30-1.14 (m, 36H), 0.81 (m, 6H). **^{13}C NMR** (101 MHz, CD_2Cl_2): δ (ppm) = 168.0, 165.2, 164.8, 163.6, 163.5, 154.6, 154.4, 151.9, 150.9, 148.6, 144.8, 141.1, 122.1, 120.6, 120.3, 117.0, 114.8, 111.4, 107.5, 87.2, 77.9, 69.7, 57.6, 39.9, 36.3, 32.3, 32.3, 31.6, 30.10, 30.06, 30.04, 30.02, 29.99, 29.97, 29.92, 29.77, 29.74, 29.66, 29.13, 28.6, 27.8. 26.1, 23.1, 18.8, 14.3; **HRMS** (ESI, positive mode, MeCN/$CHCl_3$): *m/z* calcd for $C_{51}H_{73}N_5O_6$ [M]$^+$: 851.5555, found: 851.5545.

*Building block **Bb***

Dark blue solid (101 mg, 35%). **Mp.**: 118–121 °C (from CH_2Cl_2). **^{1}H NMR** (400 MHz, CD_2Cl_2): δ (ppm) = 8.41 (t, J = 6.6 Hz, 1H), 8.02 (d, J = 7.1 Hz, 2H), 7.83-7.76 (m, 2H), 7.70 (d, J = 2.5 Hz, 1H), 7.45 (dd, J_1 = 14.4 Hz, J_2 = 11.7 Hz, 1H), 7.34 (d, J = 7.1 Hz, 2H), 7.11 (d, J = 14.1 Hz), 6.26 (d, J = 14.4 Hz), 5.81 (t, J = 6.1 Hz, 1H), 4.28 (t, J = 6.5 Hz, 2H), 4.14 (t, J = 6.5 Hz, 2H), 4.00 (t, J = 6.0 Hz, 2H), 3.96 (s, 3H), 3.60-3.56 (m, 2H), 3.01-2.96 (m, 2H), 2.33 (s, 3H), 2.28-2.25 (m, 2H), 1.85-1.78 (m, 2H), 1.77-1.72 (m, 2H), 1.52-1.40 (m, 11H), 1.39-1.25 (m, 18H), 0.88 (t, J = 6.8 Hz, 3H). **^{13}C NMR** (101 MHz, CD_2Cl_2): δ (ppm) = 168.1, 165.2, 164.9, 164.0, 163.9, 156.3, 155.0, 154.7, 151.8, 151.0, 148.7, 144.7, 141.3, 122.3, 120.9, 120.1, 117.4, 114.8, 111.4, 107.3, 87.1, 78.6, 69.7, 57.6, 42.6, 37.5, 36.7, 36.2, 32.3, 31.8, 30.04, 30.02, 29.96, 29.92, 29.73, 29.65, 29.12, 28.7, 28.6, 26.2, 23.1, 18.9, 18.7, 17.5, 14.3, 12.2; **HRMS** (ESI, positive mode, MeCN/$CHCl_3$): *m/z* calcd for $C_{47}H_{64}N_6NaO_8$[M + Na]$^+$: 863.4683, found: 863.4671.

Building block ***Bc***

Dark blue solid (604 mg, 45%). **Mp.**: 108–110 °C (from CH_2Cl_2). **^{1}H NMR** (400 MHz, CD_2Cl_2): δ (ppm) = 7.87 (d, *J* = 7.0 Hz, 2H), 7.76 (dd, J_1 = 11.5 Hz, J_2 = 13.8 Hz 1H), 7.42 (dd, J_1 = 11.5 Hz, J_2 = 14.5 Hz 1H), 7.38 (d, *J* = 7.0 Hz, 2H), 7.04 (d, *J* = 13.8 Hz 1H), 6.27 (d, *J* = 14.5 Hz, 1H), 5.77 (t, *J* = 6.0 Hz, 1H), 4.20 (t, *J* = 7.4 Hz, 2H), 3.99 (t, *J* = 6.0 Hz, 2H), 3.00-2.96 (m, 2H), 2.27 (s, 3H), 1.91-1.88 (m, 2H), 1.78-1.70 (m, 2 H), 1.44 (s, 9H), 1.36-1.23 (m, 18H), 0.87 (t, *J* = 6.7 Hz, 3H). **^{13}C NMR** (101 MHz, CD_2Cl_2): *δ* (ppm) = 164.0, 163.8, 156.3, 154.6, 154.6, 150.5, 144.0, 141.1, 122.5, 121.1, 120.3, 117.8, 107.2, 86.4, 78.7, 60.4, 37.6, 36.8, 32.3, 31.4, 30.0, 29.8, 29.7, 29.3, 28.8, 28.6, 26.5, 23.1, 18.9, 14.3; **HRMS** (ESI, positive mode, MeCN/$CHCl_3$): *m/z* calcd for $C_{36}H_{52}N_4NaO_4$[M + Na]$^+$: 627.3886, found: 627.3870.

Deprotection of the type B building blocks

Deprotection of ***Ba***

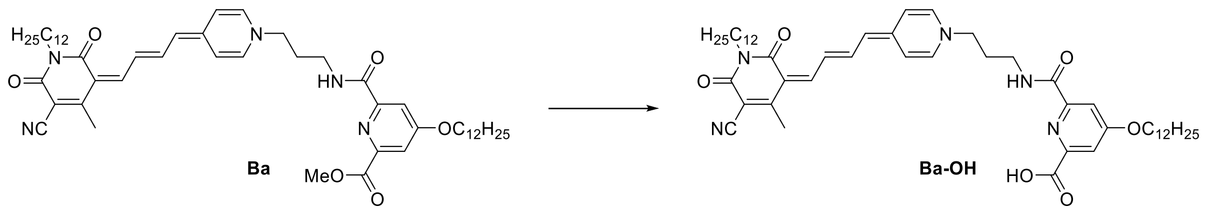

Monomer **Ba** (107 mg, 125 µmol, 1 equiv.) was dissolved in 4 mL MeOH/THF (50/50, v/v%) and 4M NaOH aqueous solution (62.5 µL, 250 µmol, 2 equiv.) was slowly added into the solution at room temperature. The mixture was stirred at room temperature for 2 h (note: the desired product slowly decomposes, therefore, the appropriate reaction time should be determined by TLC monitoring). The reaction mixture was then diluted with dichloromethane and washed with 10 mM HCl aqueous solution. The aqueous layer was extracted with 30 mL CH_2Cl_2 three times (The addition of small amounts of MeOH might be necessary to properly dissolve the product). The organic layers were combined and (without drying over $MgSO_4$ or similar) condensed under vacuum. The resulting dark blue crude product was rigorously dried under high vacuum and used for the next reaction without further purification.

Deprotection of ***Bb***

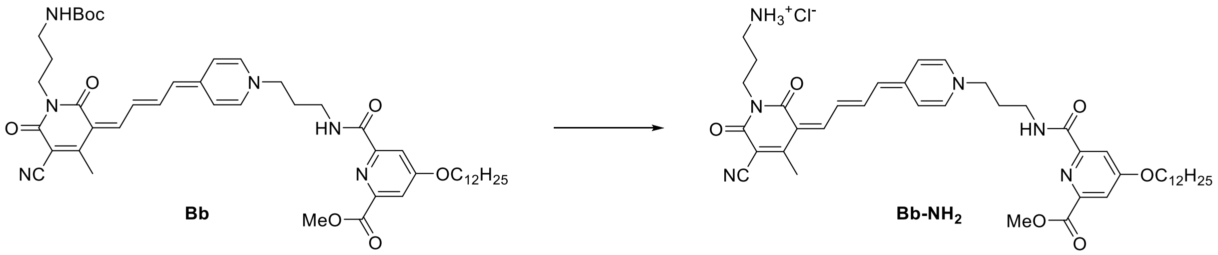

Monomer **Bb** (101 mg, 120 µmol, 1 equiv.) was dissolved in 6 mL dichloromethane and 4 M HCl in 1,4-dioxane (0.3 mL, 1.20 mmol, 10 equiv.) was slowly added into the dichloromethane solution at room temperature. The mixture was stirred at room temperature for 1 h (note: the desired product slowly decomposes, therefore, the appropriate reaction time should be determined by TLC monitoring; moreover, if precipitation occurs, small amounts of methanol should be added). The solvent was evaporated under vacuum as fast as possible and the residue immediately precipitated in diethyl ether to get rid of remaining HCl and solvents. The resulting dark blue crude product was rigorously dried under high vacuum and used for the next reaction without further purification.

*Deprotection of **Bc***

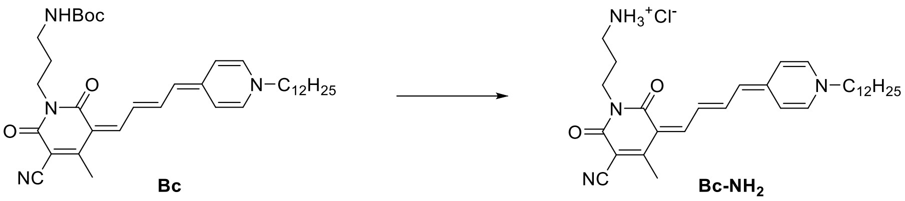

Bc **Bc-NH₂**

Monomer **Bc** (604 mg, 999 µmol, 1 equiv.) was dissolved in 30 mL dichloromethane and 4 M HCl in 1,4-dioxane (2.5 mL, 9.99 mmol, 10 equiv.) was slowly added into the dichloromethane solution at room temperature. The mixture was stirred at room temperature for 1 h (note: the desired product slowly decomposes, therefore, the appropriate reaction time should be determined by TLC monitoring; moreover, if precipitation occurs, small amounts of methanol should be added). The solvent was evaporated under vacuum as fast as possible and the residue immediately precipitated in diethyl ether to get rid of remaining HCl and solvents. The resulting dark blue crude product was rigorously dried under high vacuum and used for the next reaction without further purification.

General Procedure for the synthesis of the intermediate dimer

The thoroughly dried acid **Ba-OH** or **Ra-OH** (1 equiv.), amine **Bb-NH$_2$** or **Rb-NH$_2$** (1.5 equiv.) and HBTU (2 equiv.) were suspended in anhydrous dichloromethane, followed by addition of Hünig's base (5 equiv.). The reaction mixture was stirred at room temperature for 1 h under nitrogen atmosphere. The solvent was evaporated under vacuum and the crude product was washed with 10 mL methanol three times. If necessary further purification was carried out by size-exclusion chromatography (SX1, CH_2Cl_2/MeOH = 9:1) and precipitation from methanol.

RB-OMe

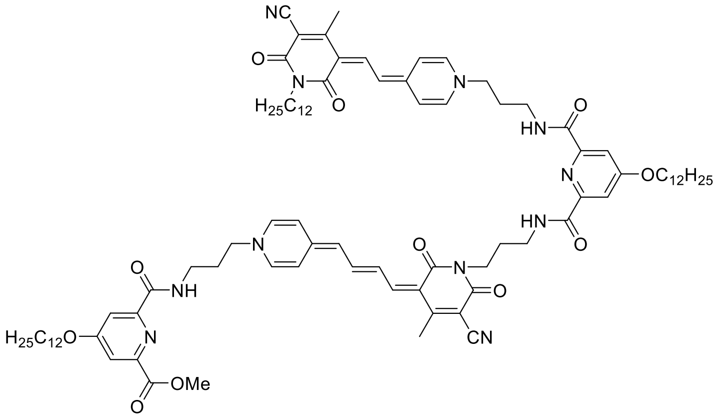

Purple solid that turns brown upon drying (75%); **Mp.**: 141-143 °C (from CH_2Cl_2). **^{1}H NMR** (400 MHz, $CDCl_3$): δ (ppm) = 9.88 (t, *J* = 6.6 Hz, 1H), 9.49 (t, *J* = 5.5 Hz, 1H) 8.78-8.73 (br, 1H), 8.28 (br, 2H), 7.84 (d, *J* = 2.4 Hz, 1H), 7.82-7.65 (m, 7H) 7.43-7.36 (m, 3H), 7.18 (d, *J* = 7.1 Hz, 2H), 7.10 (m, 1H), 6.78 (d, *J* = 14.5 Hz, 1H), 6.31 (d, *J* = 14.7 Hz, 1H) 4.41-4.34 (br, 2H), 4.28-4.24 (br, 2H), 4.16-4.11 (m, 4H), 4.02-3.96 (m, 5H), 3.85-3.76 (br, 2H), 3.70-3.63 (br, 2H) 2.61-2.53 (br, 2H), 2.45-2.37 (br, 4H), 2.23 (s, 6H), 2.02-1.93 (br, 2H), 1.87-1.79 (m, 4H), 1.66-1.58 (m, 2H), 1.50-1.42 (m, 4H), 1.39-1.19 (m, 52H), 0.90-0.84 (m, 9H); **^{13}C-NMR** (101 MHz, $CDCl_3$): δ (ppm) = 167.8, 165.1, 165.0, 164.7, 163.6, 163.4, 163.3, 163.2, 156.9, 156.1, 154.8, 154.6, 151.9, 151.2, 150.1, 149.0, 148.0, 142.2, 141.9, 140.0, 139.4, 123.1, 121.9, 120.4, 119.3, 118.8, 115.1, 114.1, 111.2, 110.6, 110.5, 107.5, 106.8, 88.1, 85.0, 69.4, 69.1, 57.5, 55.8, 53.2, 40.1, 36.4, 36.0, 35.9, 34.5, 32.1, 31.2, 29.9, 29.83, 29.78, 29.76, 29.71, 29.68, 29.66, 29.50, 29.48, 29.42, 29.40, 28.93, 28.88, 28.5, 27.8, 27.6, 27.2, 26.0, 22.8, 18.9, 14.3; **HRMS** (ESI-TOF, positive, MeCN/$CHCl_3$): m/z calculated for $C_{90}H_{124}N_{11}O_{11}^+$ $[M+H]^+$: 1534.9482, found: 1534.9427.

BR-OMe

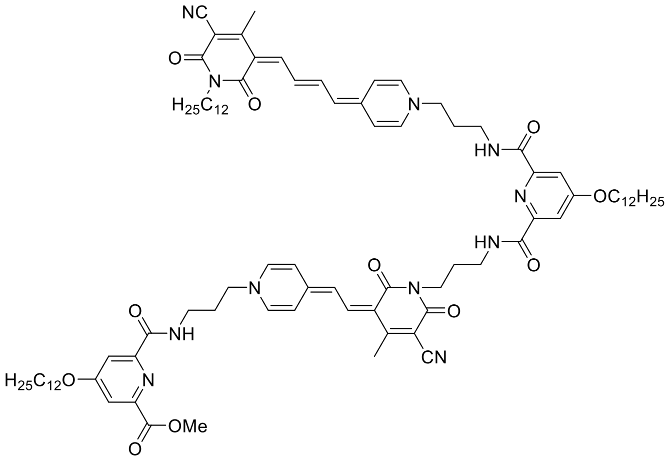

Purple solid that turns brown upon drying (75%); **Mp.**: 258-260 °C (from CH_2Cl_2). **^{1}H NMR** (400 MHz, $CDCl_3$): δ (ppm) = 9.54 (t, *J* = 6.3 Hz, 1H) 9.28 (t, *J* = 5.5 Hz, 1H), 8.60 (t, *J* = 6.4 Hz, 1H) 7.91(d, *J* = 6.8 Hz, 2H), 7.85-7.73 (m, 7H), 7.70 (d, *J* = 2.5 Hz, 1H), 7.42 (d, *J* = 15.0 Hz, 1H), 7.25 (d, 2H), 7.15 (d, *J* = 6.9 Hz, 2H), 7.05 (dd, J_1 = 11.9 Hz, J_2 = 14.5 Hz, 1H), 6.76 (d, *J* = 14.0 Hz, 1H), 6.18 (d, *J* = 14.5 Hz, 1H), 4.40 (t, *J* = 6.2 Hz, 2H), 4.28 (t, *J* = 5.4 Hz, 2H), 4.17-4.10 (m, 4H), 4.00 (s, 3H), 3.96-3.91 (m, 2H), 3.76-3.66 (br, 2H), 3.59-3.51 (m, 2H), 2.71-2.45 (br, 2H), 2.29-2.21 (m, 5H), 2.12-2.05 (m, 7H), 1.87-1.79 (m, 4H), 1.63-1.53 (m, 2H), 1.49-1.42 (m, 4H), 1.39-1.14 (m, 56H), 0.90-0.84 (m, 9H); **^{13}C-NMR** (101 MHz, $CDCl_3$): δ (ppm) = 168.2, 167.9, 165.9, 165.0, 164.65, 164.59, 163.6, 163.35, 163.29, 163.1, 156.6, 156.4, 154.4, 154.3, 151.4, 151.0, 150.1, 148.0, 144.4, 141.1, 140.4, 139.4, 122.1, 120.9, 120.5, 119.8, 119.7, 117.0, 116.1, 115.2, 115.1, 111.1, 110.7, 107.5, 106.7, 87.1, 86.3, 69.4, 69.1, 57.6, 56.1, 53.3, 53.2, 40.1, 36.9, 35.9, 35.8, 34.8, 32.1, 31.1, 29.9, 29.84, 29.79, 29.77, 29.72, 29.67, 29.52, 29.49, 29.42, 28.9, 28.8, 28.5, 27.8, 27.5, 27.2, 26.0, 22.8, 18.9, 18.8, 14.3; **HRMS** (ESI-TOF, positive, MeCN/$CHCl_3$): m/z calculated for $C_{90}H_{123}N_{11}O_{11}^+$ $[M]^+$: 1533.9398, found: 1533.9346.

BB-OMe

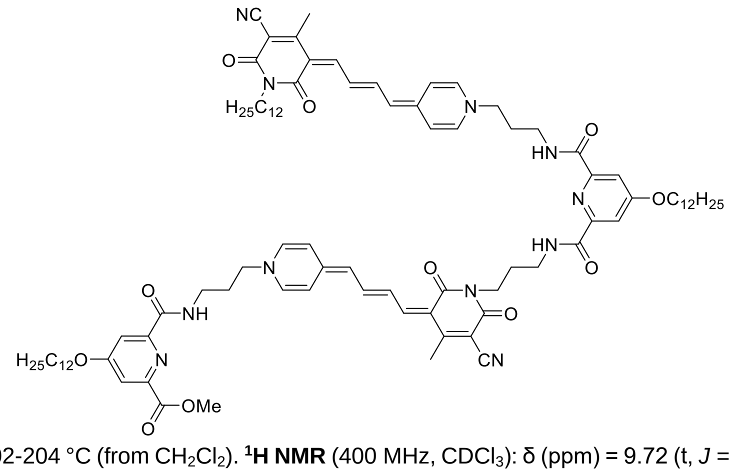

Dark blue solid (64%). **Mp.**: 202-204 °C (from CH_2Cl_2). **^{1}H NMR** (400 MHz, $CDCl_3$): δ (ppm) = 9.72 (t, *J* = 6.6 Hz, 1H) 9.45 (t, *J* = 5.4 Hz, 1H), 8.76 (t, *J* = 6.0 Hz, 1H), 8.12(d, *J* = 6.7 Hz, 2H), 7.84-7.72 (m, 7H), 7.70 (d, *J* = 2.4 Hz, 1H), 7.23 (d, *J* = 6.9 Hz, 2H), 7.15-7.06 (m, 4H), 6.82 (d, *J* = 14.3 Hz, 1H), 6.76 (d, *J* = 14.0 Hz, 1H), 6.23 (d, *J* = 14.7 Hz, 1H), 6.18 (d, *J* = 14.5 Hz, 1H), 4.41 (t, *J* = 6.4 Hz, 2H), 4.28 (t, *J* = 5.8 Hz, 2H), 4.17-4.10 (m, 4H), 4.00 (s, 3H), 3.96-3.91 (m, 2H), 3.83-3.71 (br, 2H), 3.67-3.61 (m, 2H), 2.61-2.50 (br, 2H), 2.44-2.36 (m, 2H), 2.24-2.15 (m, 5H), 2.07-1.94 (m, 5H), 1.87-1.78 (m, 4H), 1.63-1.56 (m, 2H), 1.49-1.41 (m, 4H), 1.39-1.20 (m, 52H), 0.90-0.84 (m, 9H); **^{13}C-NMR** (101 MHz, $CDCl_3$): δ (ppm) = 168.1, 167.7, 165.4, 165.1, 164.8, 164.6, 163.3, 154.5, 154.1, 154.0, 151.8, 151.1, 150.5, 150.1, 149.2, 148.0, 144.5, 142.8, 141.7, 139.9, 123.3, 122.5, 121.0, 120.3, 119.9, 118.6, 117.2, 115.0, 111.1, 110.62, 110.57, 107.4, 107.2, 86.4, 85.4, 69.3, 69.1, 57.7, 55.9, 53.2, 40.0, 36.6, 36.1, 35.6, 34.5, 32.1, 32.0, 30.9, 29.9, 29.82, 29.78, 29.75, 29.70, 29.66, 29.50, 29.47, 29.41, 28.92, 28.88, 28.6, 27.6, 27.5, 27.2, 26.0, 25.9, 22.8, 18.9, 18.7, 14.3; **HRMS** (ESI-TOF, positive, MeCN/$CHCl_3$): m/z calculated for $C_{92}H_{126}N_{11}O_{11}^+$ $[M+H]^+$: 1560.9638, found: 1560.9520.

General procedure for the deprotection of the dimers

The respective dimer (1 equiv.) was dissolved in MeOH/THF (66/33, v/v%) and 1M NaOH aqueous solution (2 equiv.) was slowly added into the solution at room temperature. The mixture was stirred at room temperature for 2 h (note: the desired product slowly decomposes, therefore, the appropriate reaction time should be determined by TLC monitoring). The reaction mixture was then diluted with dichloromethane and washed with 10 mM HCl aqueous solution. The aqueous layer was extracted with CH_2Cl_2 three times (The addition of small amounts of MeOH might be necessary to dissolve the product). The organic layers were combined and (without drying over $MgSO_4$ or similar) condensed under vacuum. The resulting crude product was rigorously dried under high vacuum and used for the next reaction without further purification.

General procedure for the synthesis of the trimers

The respective deprotected dimer (1 equiv), amine **Rc-NH₂** or **Bc-NH₂** (1.5 equiv.) and HBTU (2 equiv.) were suspended in anhydrous dichloromethane, followed by addition of Hünig's base (5 equiv.). The reaction mixture was stirred at room temperature for 1 h under nitrogen atmosphere. The solvent was evaporated under vacuum. The crude product was purified column chromatography (eluent CH_2Cl_2/MeOH 94:6), size-exclusion chromatography (SX1, CH_2Cl_2/MeOH = 9:1) and subsequent precipitation from methanol and n-hexane, to yield the target compound.

RBR

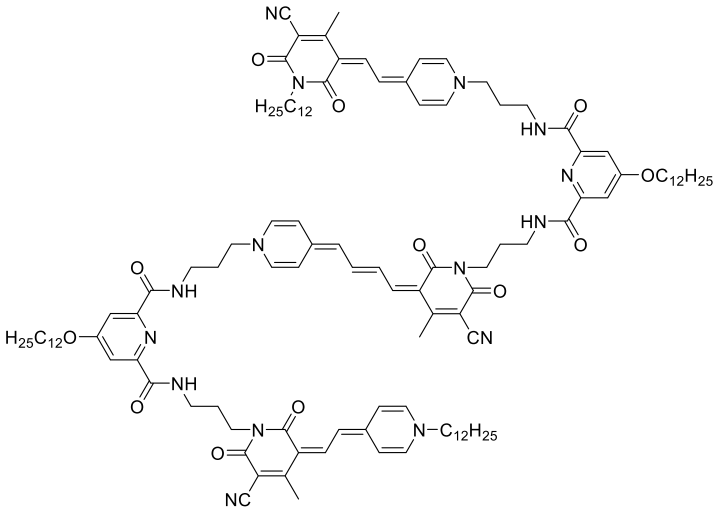

Dark purple solid (59.2 mg, 45%); **Mp.**: 179–181 °C (from CH_2Cl_2). **^{1}H NMR** (400 MHz, $CDCl_3$): δ (ppm) = 9.85 (t, J = 6.2 Hz,1 H), 9.69 (t, J = 6.6 Hz,1 H), 9.53 (t, *J* = 5.7 Hz,1 H), 9.41 (t, *J* = 5.9 Hz,1 H), 8.17 (d, *J* = 6.6 Hz, 2H), 7.92-7.69 (m, 12H), 7.52-7.47 (m, 3H), 7.37 (d, *J* = 6.6 Hz, 2H), 7.22-7.16 (m, 3H), 6.87 (d, *J* = 14.2 Hz, 1H), 6.25 (d, *J* = 14.7 Hz, 1H), 4.41 (t, *J* = 6.4 Hz, 2H) 4.29-4.15 (m, 8H), 4.12 (t, *J* = 6.4 Hz, 4H), 3.96-3.92 (m, 2H), 3.75-3.71 (m, 4H), 3.59-3.04 (br, 2H), 2.52-2.45 (m, 7H), 2.34 (s, 3H), 2.27 (s, 3H), 2.06-1.97 (br, 4H), 1.89-1.77 (m, 6H), 1.63-1.55 (m, 2H), 1.48-1.39 (m, 4H), 1.38-1.18 (m, 70H), 0.89-0.83 (m, 12H); **^{13}C-NMR** (101 MHz, $CDCl_3$): δ (ppm) = 168.1, 165.2, 164.9, 164.0, 163.9, 156.3, 155.0, 154.7, 151.8, 151.0, 148.7, 144.7, 141.3, 122.3, 120.9, 120.1, 117.4, 114.8, 111.4, 107.3, 87.1, 78.6, 69.7, 57.6, 42.6, 37.5, 36.7, 36.2, 32.3, 31.9, 30.0, 30.0, 30.0, 29.9, 29.7, 29.7, 29.1, 28.7, 28.6, 26.2, 23.1, 18.9, 18.7, 17.5, 14.3, 12.2; **HRMS** (ESI-TOF, positive, MeCN/$CHCl_3$): m/z calculated for $C_{118}H_{161}N_{15}Na_2O_{12}^{2+}$ $[M+2Na]^{2+}$: 1013.1117, found: 1013.1122; **UV/Vis** ($CHCl_3$): λ_{max} = 518 nm (ε = 158000 $M^{-1}cm^{-1}$).

RRB

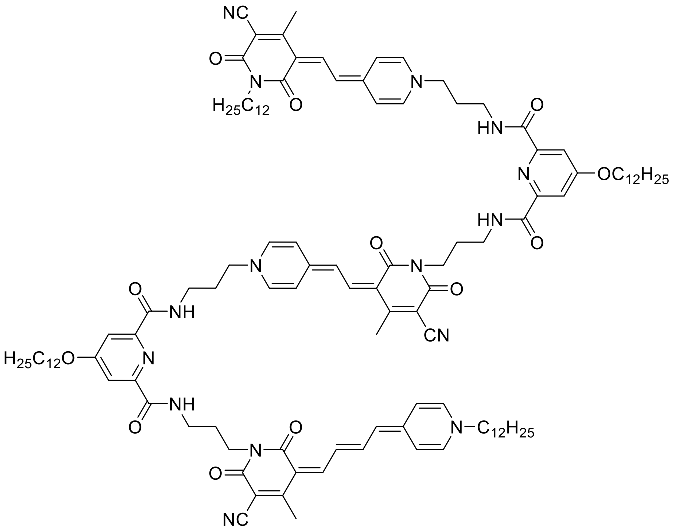

Dark purple solid (29.5 mg, 37%); **Mp.**: 183-186 °C (from CH_2Cl_2). **^{1}H NMR** (400 MHz, $CDCl_3$): δ (ppm) = 9.85-9.75 (m, 2H), 9.54-9.45 (m, 2H), 7.99 (d, *J* = 6.3 Hz, 2H), 7.85-7.67 (m, 11H), 7.43-7.28 (m, 5H), 7.22 (d, *J* = 6.4 Hz, 2H), 7.16 (d, *J* = 6.7 Hz,2H), 6.95 (d, *J* = 14.2 Hz, 1H), 6.32 (d, *J* = 14.7 Hz, 1H), 4.41 (t, *J* = 6.5 Hz, 2H) 4.25-4.10 (m, 10H), 3.96-3.92 (m, 2H), 3.91-3.85 (m, 2H), 3.76-3.68 (m, 4H), 3.51-3.11 (br, 2H), 2.56-2.42 (m, 4H), 3.46-3.16 (br, 2H), 2.32 (s, 3H), 2.29 (s, 3H), 2.22 (s, 3H), 2.06-1.94 (br, 4H), 1.87-1.78 (m, 4H), 1.75-1.66 (m, 2H), 1.59-1.52 (m, 2H), 1.48-1.41 (m, 4H), 1.39-1.18 (m, 70H), 0.89-0.83 (m, 12H).; **^{13}C-NMR** (101 MHz, $CDCl_3$): δ (ppm) = 168.3; 168.1; 165.1, 164.9, 164.84, 164.78, 163.6, 163.4, 163.3, 163.2, 163.1, 156.8, 156.5, 155.9, 154.9, 154.5, 151.0, 150.22, 150.17, 149.9, 143.1, 141.2, 140.8, 139.8, 138.0, 123.2, 121.5, 120.5, 120.2, 119.5, 118.5, 115.3, 113.5, 110.7, 107.7, 106.7, 106.5, 88.6, 86.3, 85.8, 69.3, 69.1, 59.9, 57.1, 56.0, 39.9, 36.7, 36.3, 36.0, 34.7, 32.0, 32.0, 31.2, 29.9, 29.83, 29.79, 29.76, 29.72, 29.68, 29.59, 29.51, 29.48, 29.46, 29.44, 29.0, 28.9, 28.5, 27.5, 26.2, 26.0, 22.82, 22.81, 18.9, 18.8, 18.7, 14.3; **HRMS** (ESI-TOF, positive, MeCN/$CHCl_3$): m/z calculated for $C_{118}H_{161}N_{15}Na_2O_{12}^{2+}$ $[M+2Na]^{2+}$: 1013.1117, found: 1013.1127; **UV/Vis** ($CHCl_3$): λ_{max} = 483 nm (ε = 136000 $M^{-1}cm^{-1}$).

BRR

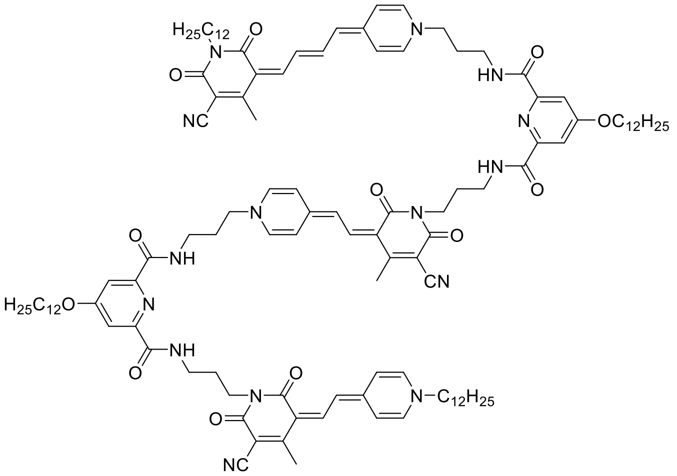

Dark purple solid (85 mg, 63%); **Mp.**: 175-177 °C (from CH_2Cl_2). **^{1}H NMR** (400 MHz, $CDCl_3$): δ (ppm) = 9.72-9.62 (m, 2H), 9.45-9.37 (m, 2H), 7.95 (d, *J* = 6.5 Hz, 2H), 7.92-7.67 (m, 11H), 7.56 (d, *J* = 15.0 Hz, 1H), 7.39 (d, *J* = 6.0 Hz, 2H), 7.32 (d, *J* = 15.1 Hz, 1H), 7.22 (d, *J* = 6.6 Hz, 2H), 7.18-7.06 (m, 3H), 6.79 (d, *J* = 14.2 Hz, 1H), 6.12 (d, *J* = 14.5 Hz, 1H), 4.40 (br, *J* = 6.5 Hz, 2H), 4.32-4.08 (m, 10H), 4.03 (br, 2H), 3.91-3.85 (br, 2H), 3.74-3.61 (m, 4H), 3.51-3.11 (br, 2H), 2.53-2.41 (m, 4H), 2.35 (s, 3H), 2.22 (s, 3H), 2.14-2.02 (m, 7H), 2.02-1.93 (m, 4H), 1.86-1.77 (m, 4H), 1.77-1.69 (m, 2H), 1.57 (br, 2H), 1.49-1.39 (m, 4H), 1.39-1.18 (m, 68H), 0.89-0.83 (m, 12H).; **^{13}C-NMR** (101 MHz, $CDCl_3$): δ (ppm) = 168.3, 168.1, 165.2, 164.9, 164.72, 164.66, 163.6, 163.4, 163.2, 163.1, 157.1, 156.5, 156.1, 154.1, 151.0, 150.9, 150.2, 150.1, 144.1, 141.0, 140.7, 139.4, 138.4, 122.3, 120.9, 120.6, 119.9, 119.8, 117.2, 115.4, 114.8, 110.9, 110.8, 110.5, 107.6, 106.8, 106.6, 87.5, 86.3, 69.2, 69.1, 59.7, 57.3, 56.7, 40.1, 36.9, 36.3, 36.0, 34.8, 34.6, 32.05, 32.03, 32.01, 31.1, 29.9, 29.82, 29.78, 29.75, 29.70, 29.67, 29.57, 29.50, 29.47, 29.45, 29.43, 29.0, 28.9, 28.5, 27.5, 27.4, 27.3, 26.2, 26.0, 22.8, 18.9, 18.7, 14.3; **HRMS** (ESI-TOF, positive, MeCN/$CHCl_3$): m/z calculated for $C_{118}H_{161}N_{15}Na_2O_{12}^{2+}$ $[M+2Na]^{2+}$: 1013.1117, found: 1013.1154; **UV/Vis** ($CHCl_3$): λ_{max} = 480 nm (ε = 122000 $M^{-1}cm^{-1}$).

BBB

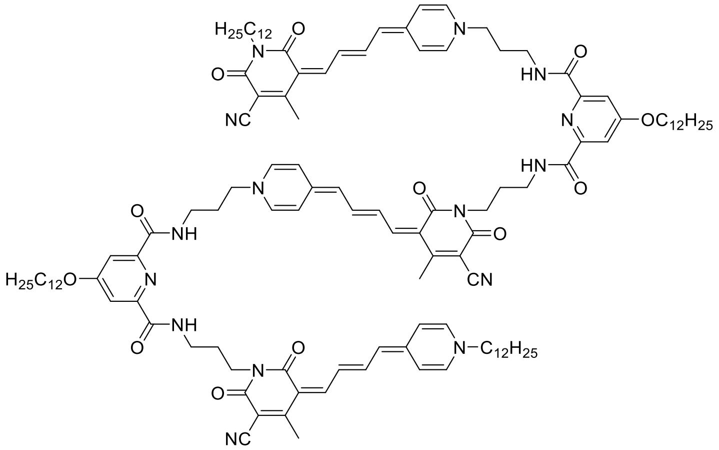

Dark blue solid (17.9 mg, 32%). **Mp.**: 197–202 °C (from CH_2Cl_2). **^{1}H NMR** (400 MHz, $CDCl_3$): δ (ppm) = 9.98 (br, 1H), 9.69 (t, *J* = 6.2 Hz, 1H), 9.63 (br, 1H), 9.45 (t, *J* = 5.5 Hz, 1H), 7.93-7.67 (m, 11H), 7.60 (d, *J* = 5.5 Hz), 7.29-7.21 (m, 3H), 7.12-6.85 (m, 7H), 6.73 (d, *J* = 12.3 Hz, 1H), 6.61 (d, *J* = 13.9 Hz), 6.24 (d, *J* = 14.7 Hz,1H), 6.17 (d, *J* = 15.1 Hz,1H), 6.03 (d, *J* = 14.3 Hz,1H), 4.49-4.43 (br, 2H), 4.31-4.18 (m, 4H), 4.17-4.11 (m, 4H), 3.94-3.88 (br, 2H), 3.87-3.81 (br, 2H), 3.79-3.65 (br, 2H), 2.57-2.43 (m, 4H), 2.20 (s, 3H), 2.07-1.97 (m, 6H), 1.89-1.79 (m, 4H), 1.62-1.51 (m, 6H), 1.50-1.43 (m, 4H), 1.36-1.11 (m, 74H), 1.10-1.04 (br, 4H), 0.90-0.85 (m, 12H); **^{13}C-NMR** (101 MHz, $CDCl_3$): δ (ppm) = 168.4, 168.1, 165.2, 164.8, 164.7, 164.6, 163.5, 163.4, 163.3, 163.0, 154.9, 154.1, 153.8, 153.7, 153.6, 151.0, 150.9, 150.4, 150.2, 148.4, 145.2, 143.9, 141.3, 141.7, 140.7, 139.5, 136.4, 123.6, 123.1, 122.3, 120.9, 120.0, 119.8, 118.9, 118.0, 116.5, 111.0, 110.9, 110.6, 107.7, 107.6, 107.3, 87.1, 86.3, 84.1, 77.4, 69.3, 69.1, 59.9, 57.1, 55.8, 39.9, 37.0, 36.8, 35.9, 35.6, 34.5, 32.1, 32.0, 30.9, 29.87, 29.83, 29.81, 29.78, 29.73, 29.70, 29.55, 29.50, 29.45, 29.0, 28.6, 27.6, 26.1, 26.02, 26.00, 22.8, 18.93, 18.91, 18.6, 14.3; **HRMS** (ESI-TOF, positive, MeCN/$CHCl_3$): m/z calculated for $C_{122}H_{165}N_{15}Na_2O_{11}$ $[M+2Na]^{2+}$: 1039.1273, found: 1039.1287. **UV/Vis** ($CHCl_3$): λ_{max} = 519 nm (ε = 185000 $M^{-1}cm^{-1}$).

2D NMR Studies

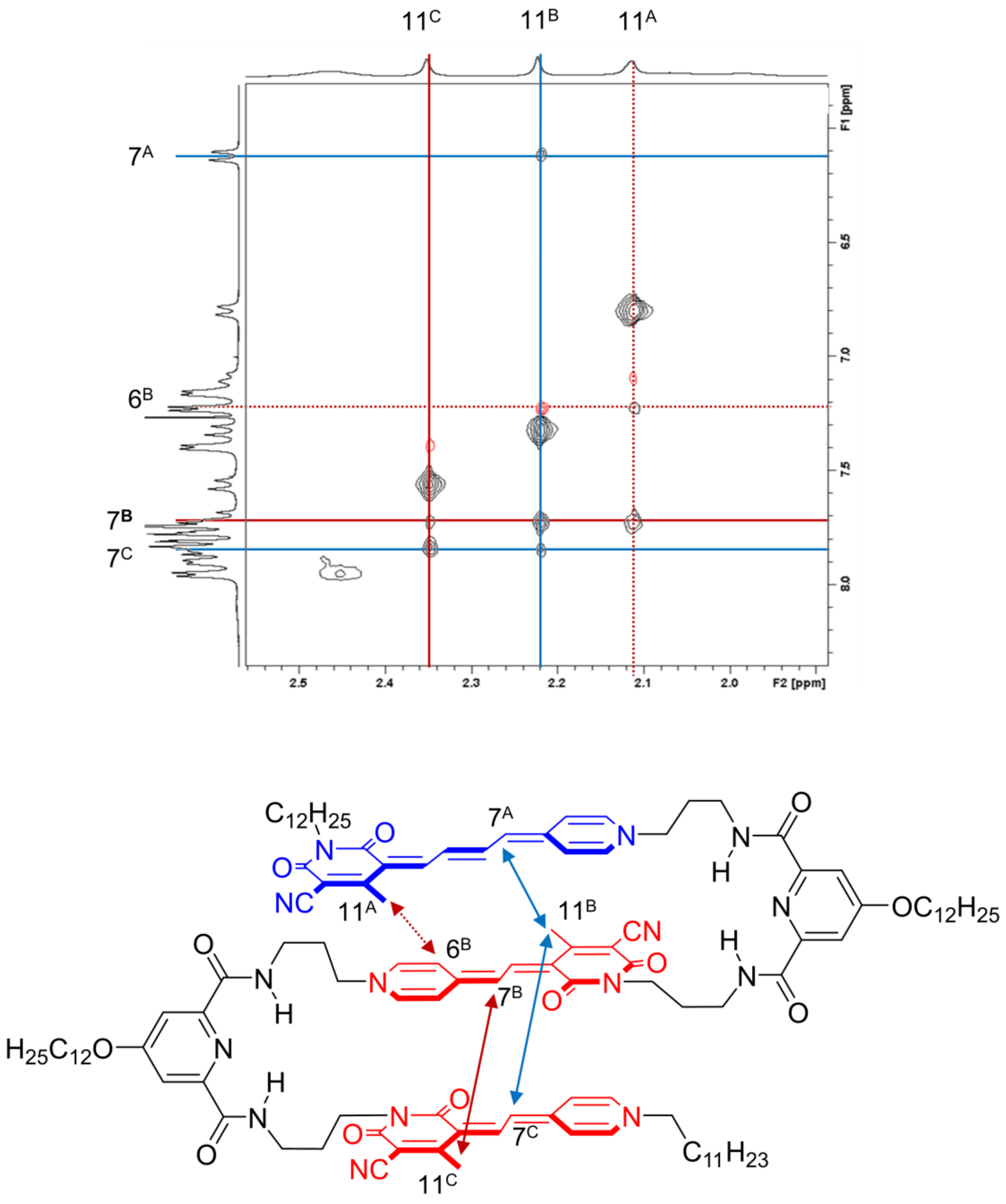

Figure S2. Excerpt of the ^{1}H-^{1}H ROESY spectrum (400 MHz) of **BRR** in $CDCl_3$ at 295 K. Also shown is the chemical structure of **BRR** with significant correlations between protons indicated by double-headed arrows.

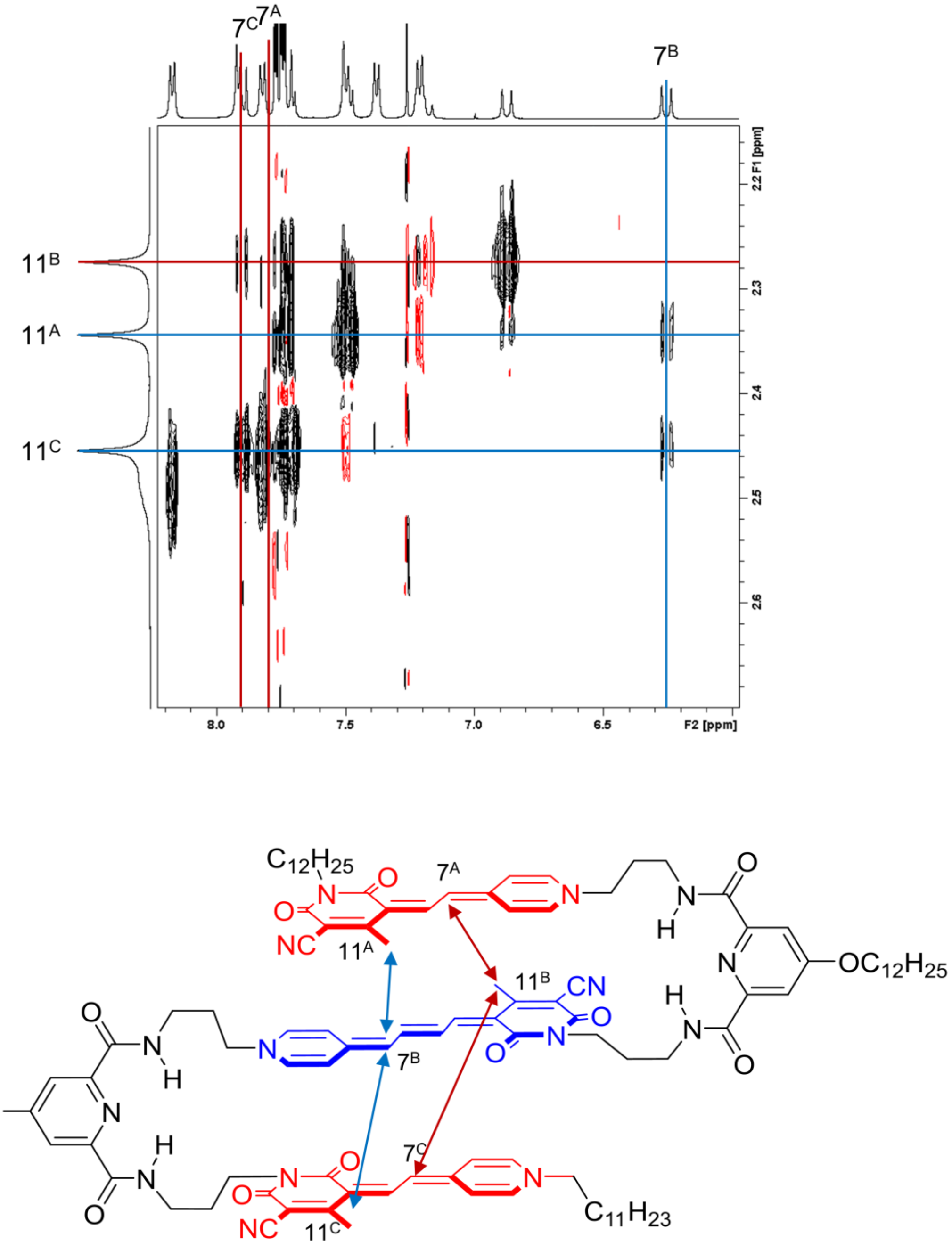

Figure S3. Excerpt of the ^{1}H-^{1}H ROESY spectrum (400 MHz) of **RBR** in $CDCl_3$ at 295 K. Also shown is the chemical structure of **RBR** with significant correlations between protons indicated by double-headed arrows.

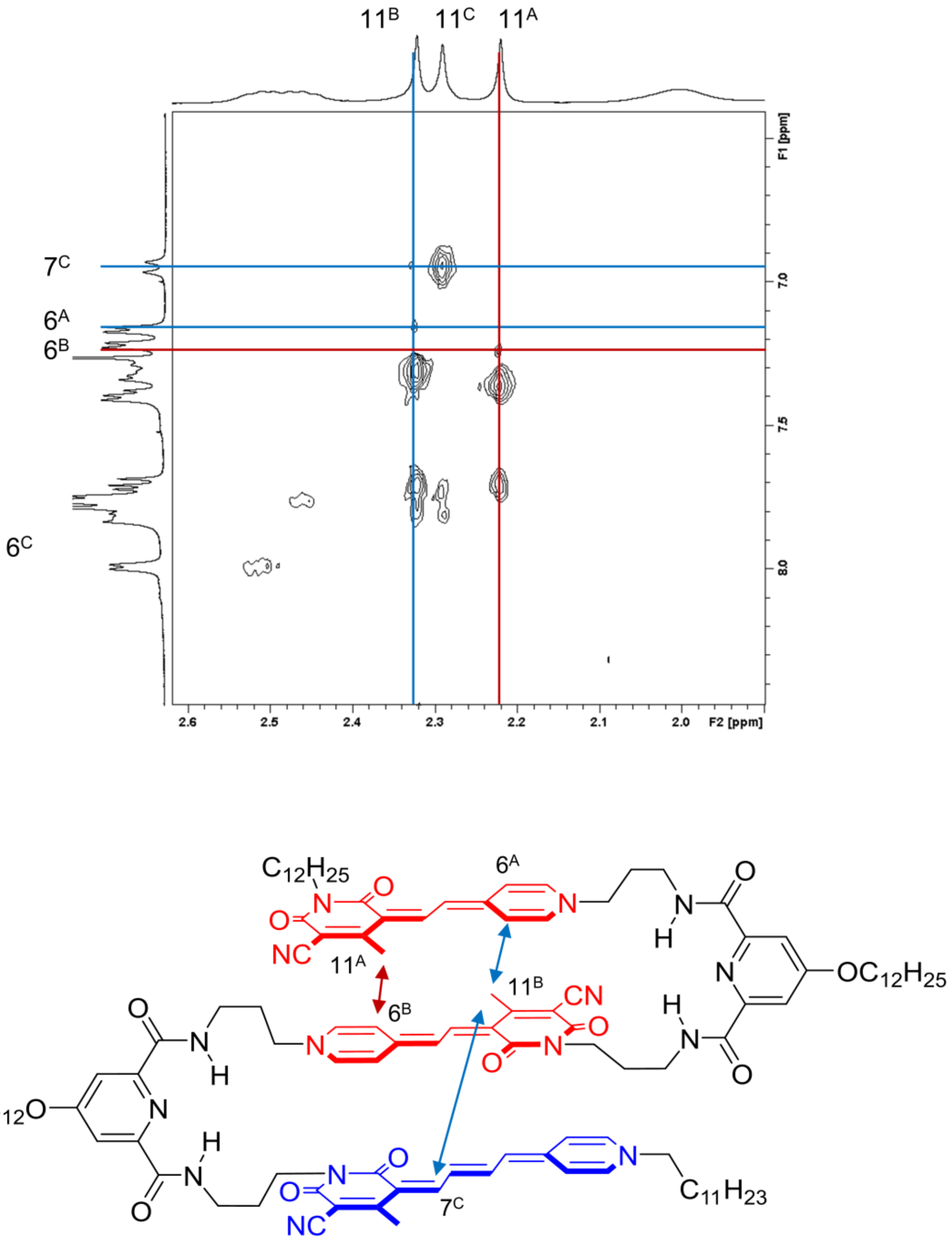

Figure S4. Excerpt of the ${}^{1}H$-${}^{1}H$ ROESY spectrum (400 MHz) of **RRB** in $CDCl_3$ at 295 K. Also shown is the chemical structure of **RRB** with significant correlations between protons indicated by double-headed arrows.

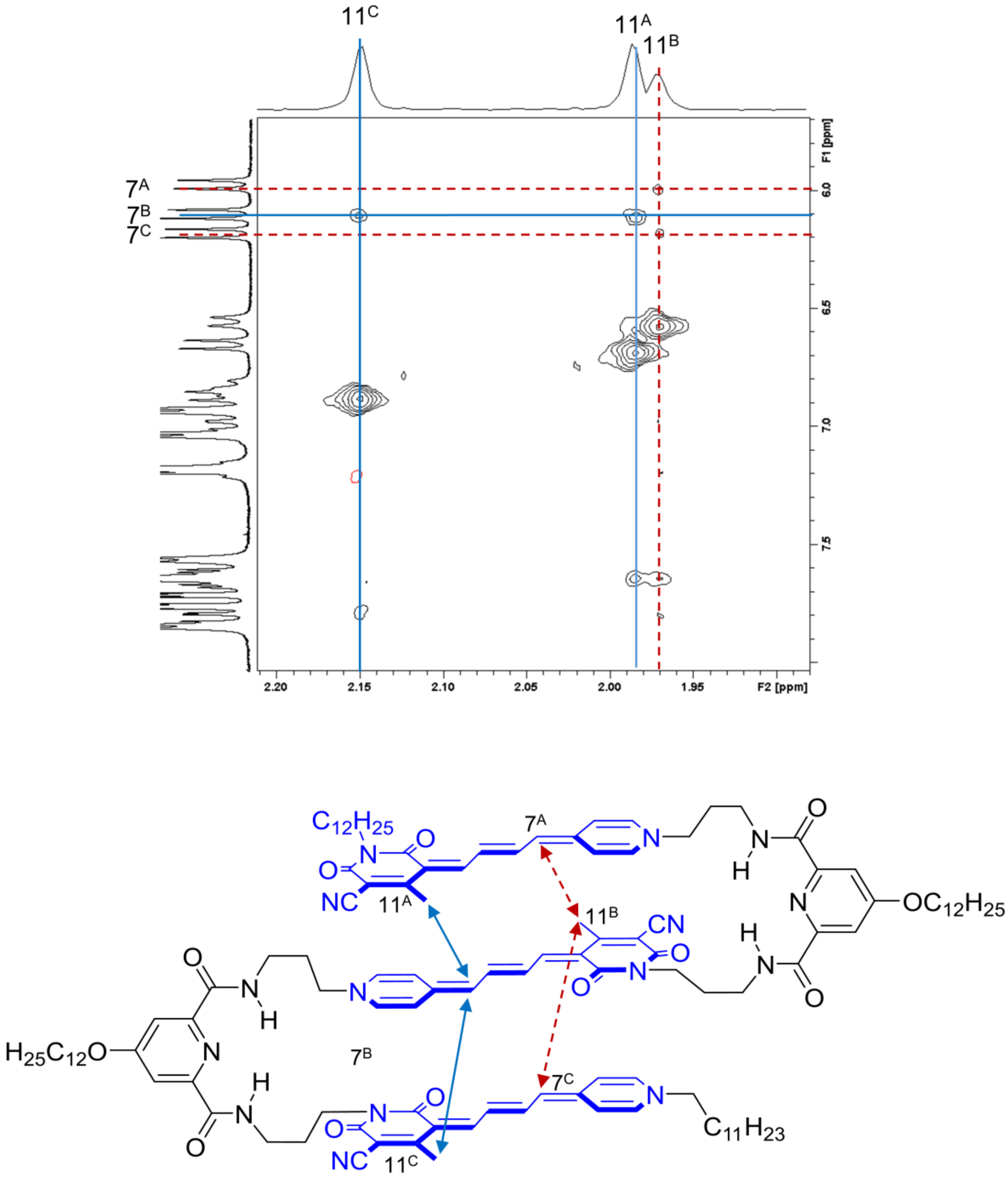

Figure S5. Excerpt of the ^{1}H-^{1}H ROESY spectrum (400 MHz) of **BBB** in $CDCl_3$ at 295 K. Also shown is the chemical structure of **BBB** with significant correlations between protons indicated by double-headed arrows.

UV-vis and fluorescence studies

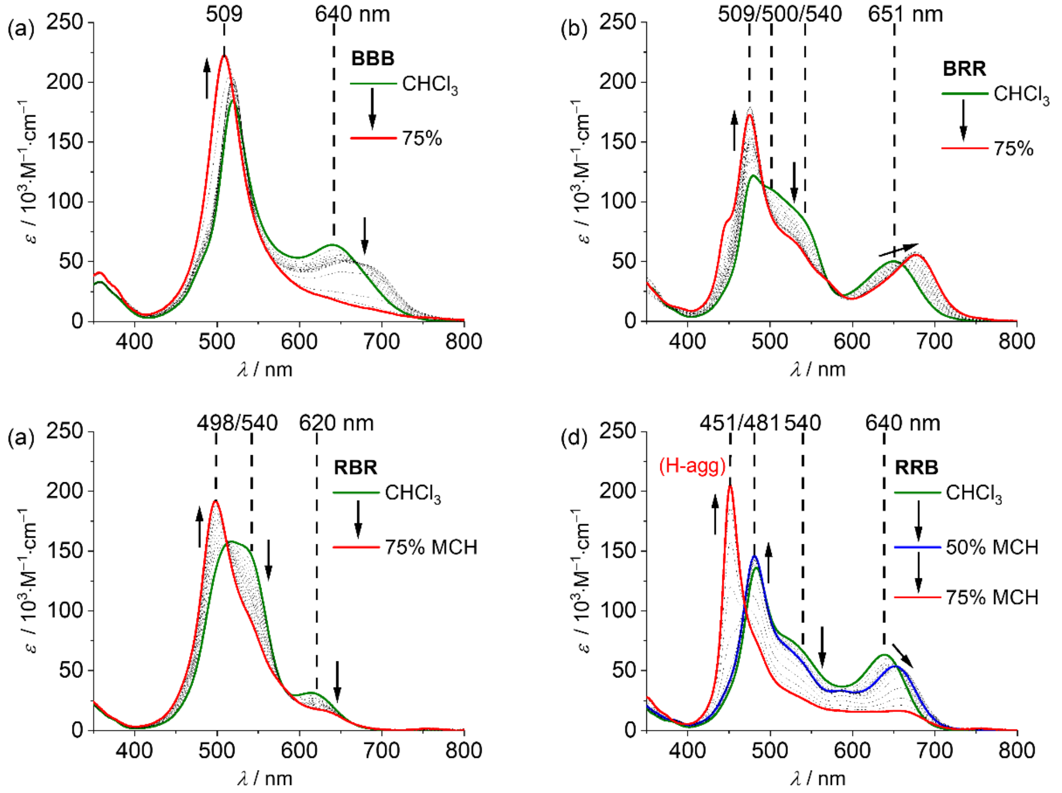

Figure S6. Solvent-dependent UV-vis spectra of MC trimers (a) **BBB**, (b) **BRR**, (c) **RBR** and(d) **RRB**. Samples were dissolved in pure $CHCl_3$ and then successively diluted with MCH to $CHCl_3$/MCH 25:75 at 293 K ($c \approx$ 1-4 μM).

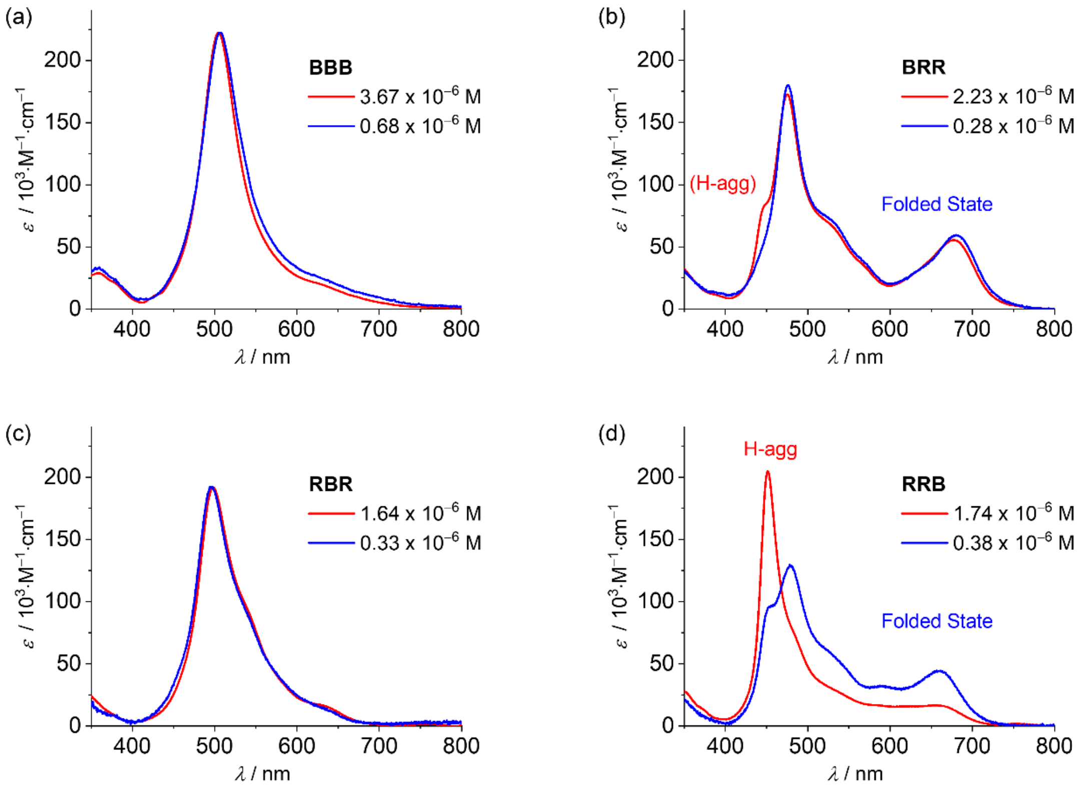

Figure S7. Concentration-dependent UV-vis spectra of MC trimers (a) **BBB**, (b) **BRR**, (c) **RBR** and (d) **RRB** in $CHCl_3$/MCH 25:75 at 293 K.

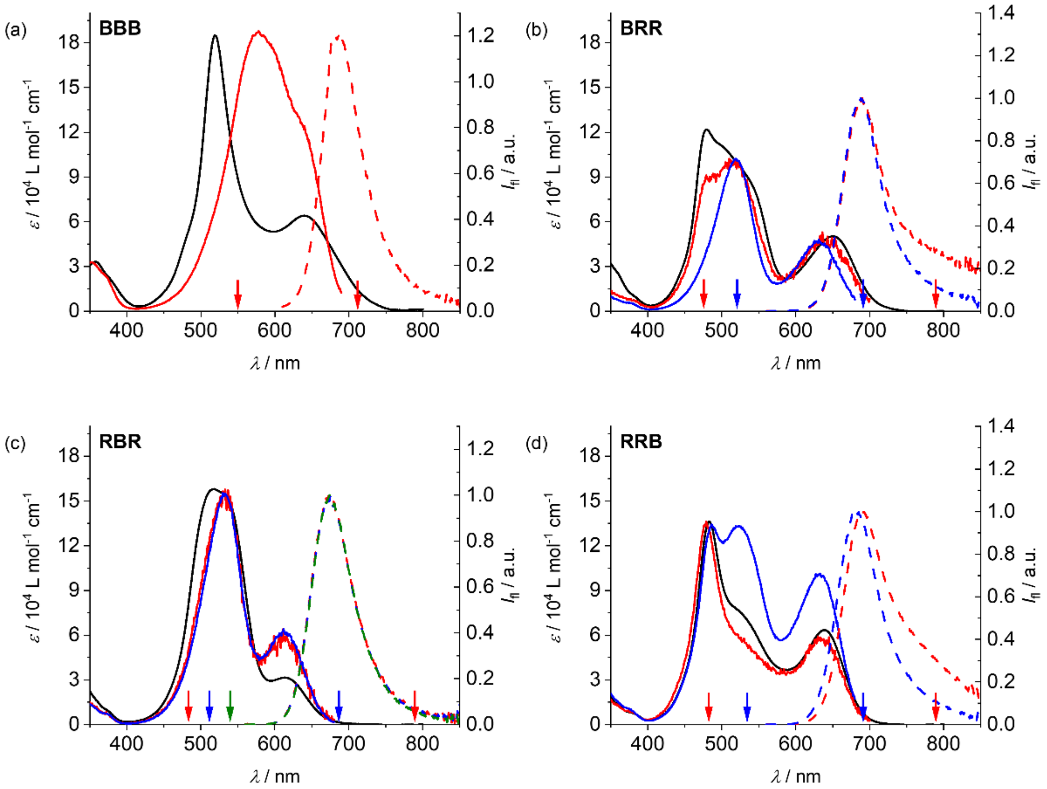

Figure S8. Normalized UV/vis absorption (black), emission (coloured dashed lines), and excitation spectra (coloured solid lines) of (a) **BBB**, (b) **BRR**, (c) **RBR** and (d) **RRB** in $CHCl_3$ at 293 K under highly dilute conditions (OD ≤ 0.05). Coloured arrows indicate the excitation wavelength or the detection wavelength of the corresponding emission or excitation spectra with the same colours.

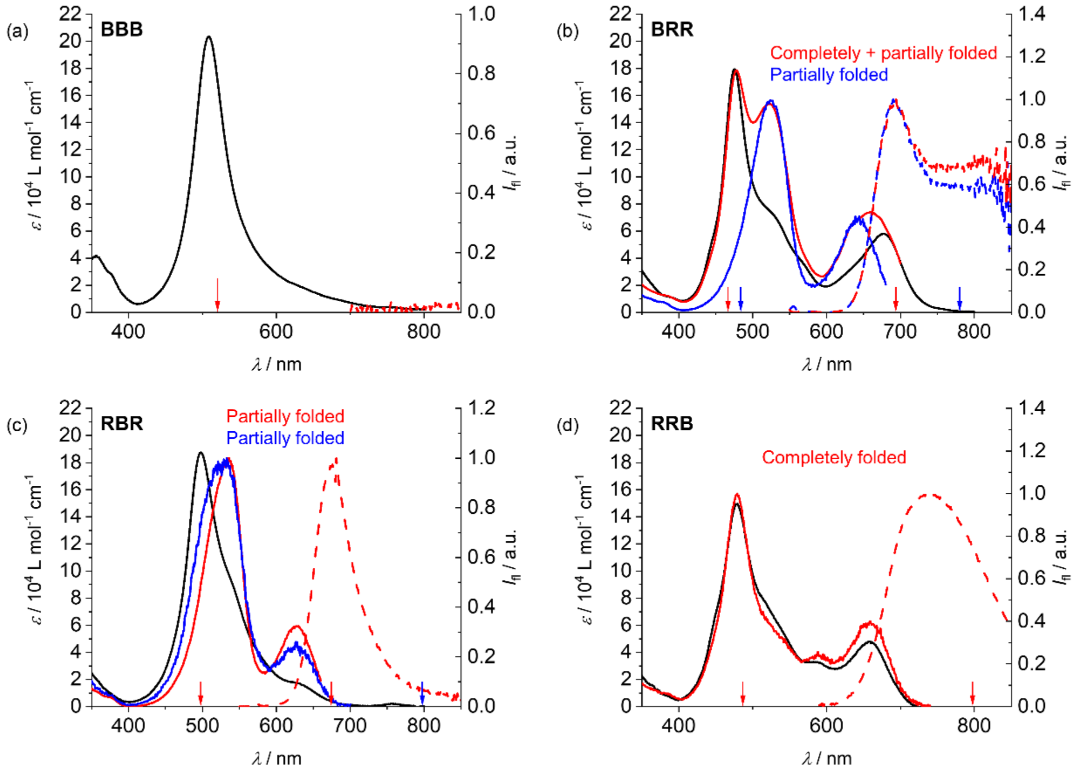

Figure S9. Normalized UV/vis absorption (black), emission (coloured dashed lines), and excitation spectra (coloured solid lines) of (a) **BBB**, (b) **BRR**, (c) **RBR** and (d) **RRB** in in $CHCl_3$/MCH 25:75 at 293 K under highly dilute conditions (OD ≤ 0.05). Coloured arrows indicate the excitation wavelength or the detection wavelength of the corresponding emission or excitation spectra with the same colours. The excitation spectra can be assigned to the form written in the same colour.

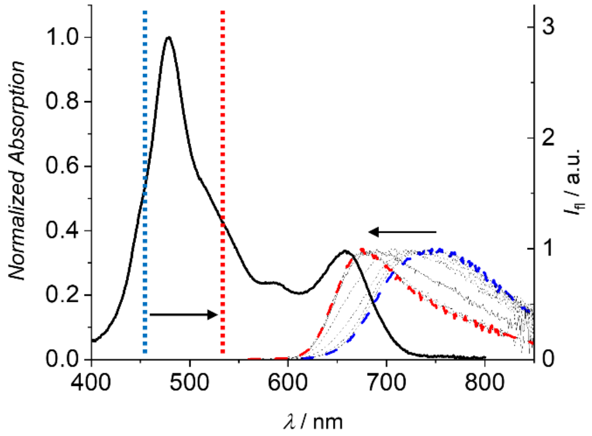

Figure S10. Absorption (solid) and emission (dashed) spectra of **RRB** in $CHCl_3$/MCH 25:75 with the excitation wavelength varied from 460 (blue) to 540 nm (red) at 293 K.

Table S3. Fluorescence data of MC references **R-ref** and **B-ref**, as well as folda-trimers in $CHCl_3$.

	Emission wavelength / nm	Life time (τ) / ns	Average life time / ns	Quantum yield (Φ_{fl})	k_r [c]/ $10^7 s^{-1}$	k_{nr} [c]/ $10^7 s^{-1}$
**R-ref**	586	0.1[d]	---	0.2%[a]	2.0	992
**B-ref**	704	0.1[d]	---	3%[b]	25	975
**BBB**	687	τ_1= 0.47 (67%)<br>τ_2= 0.88 (33%)	0.61	1.3% [b]	2.13	162
**BRR**	688	0.92	---	8.6%[a]	9.35	99
**RBR**	675	1.46	---	9.7%[a]	6.64	62
**RRB**	683	1.24	---	21.2%[a]	17.1	64

[a]Quantum yield was determined against *N*,*N*'-bis(2,6-diisopropylphenyl)-1,6,7,12-tetraphenoxy-perylene-3,4:9,10-bis(dicarboximide) (0.96 in $CHCl_3$)[S9]. [b]Quantum yield was determined against Rhodamine 800 (0.25 in Ethanol)[S10]. [c] Determined according to $k_r = \Phi/\tau$ and $k_{nr} = 1/\tau_{fl} - k_{fl}$. [d]Very short lifetime, which could not be fitted properly. The given value should be considered an approximation.

Table S4. Fluorescence data of MC reference **B-ref** and trimer **RRB** in $CHCl_3$/MCH 25:75 and $CHCl_3$/paraffin 25:75.

	Emission wavelength / nm	Solvent	Life time (τ) / ns	Average life time / ns	Quantum yield (Φ_{fl})[a]	k_r [b]/ $10^7 s^{-1}$	k_{nr} [b]/ $10^7 s^{-1}$
**B-ref**	713	$CHCl_3$/MCH	<0.1[c]	---	0.8%[d]	> 8	>992
	713	$CHCl_3$/paraffin	0.2[c]	---	2.0%[d]	10	490
**RRB**	746	$CHCl_3$/MCH	τ_1= 1.7 (86%)<br>τ_2= 3.4 (14%)	τ_{avg} = 2.0	8.9–9.8%	4.6	46
	740	$CHCl_3$/paraffin	τ_1= 1.9 (87%)<br>τ_2= 4.1 (13%)	τ_{avg} = 2.2	12.7–13.5%	6.3	39

[a] Quantum yield was determined against *N*,*N*'-bis(2,6-diisopropylphenyl)-1,6,7,12-tetraphenoxy-perylene-3,4:9,10-bis(dicarboximide (0.96 in $CHCl_3$)[S9]. [b] Determined according to $k_r = \Phi/\tau$ and $k_{nr} = 1/\tau_{fl} - k_{fl}$, [c] Very short lifetime, which could not be fitted properly. The given value should be considered as an approximation. [d] Quantum Yield of the remaining monomeric species, the self-assembled dimer is entirely quenched.

TD-DFT calculations

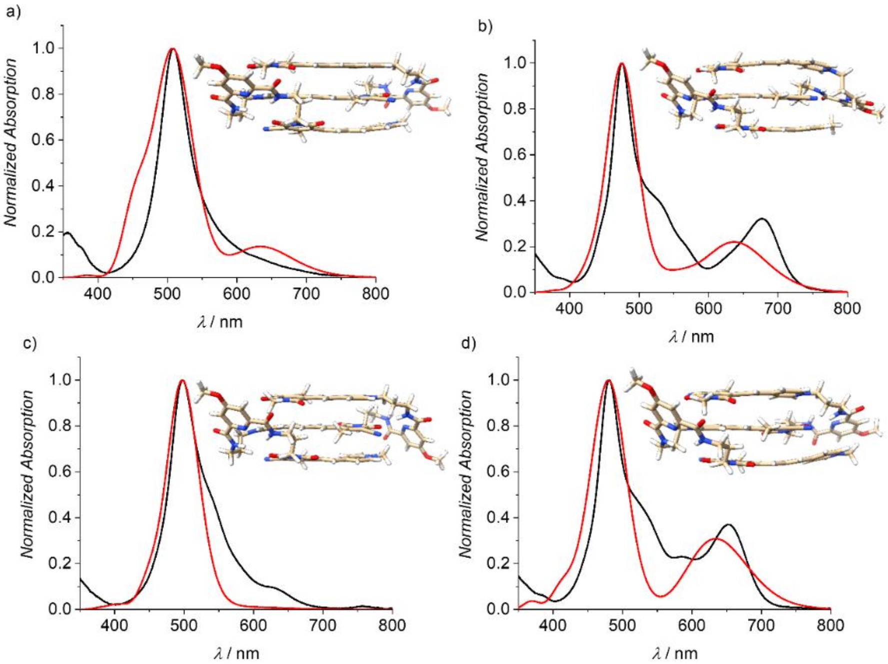

Figure S11. Calculated UV/Vis (wB97/Def2SVP) spectra (red) of (a) **BBB**, (b) **BRR**, (c) **RBR** and (d) **RRB** from TD-DFT calculations and the experimental spectra (black) in $CHCl_3$/MCH 25:75. The corresponding geometry optimized structures (B97D3/Def2SVP) are shown next to the respective spectra.

Table S5. Exciton coupling energy *J* obtained for all trimers.

Compound	Substructure	*J* [cm^{-1}][a]
**BRR**	BR	2363
	RR	1903
**RBR**	RB	2242
	BR	2168
**RRB**	RR	2322
	RB	1629
**BBB**	BB	2689
	BB	2189

[a] Calculated by transition charge method.[11] Multiwfn was used to obtain the transition charges.[12]

NMR spectra

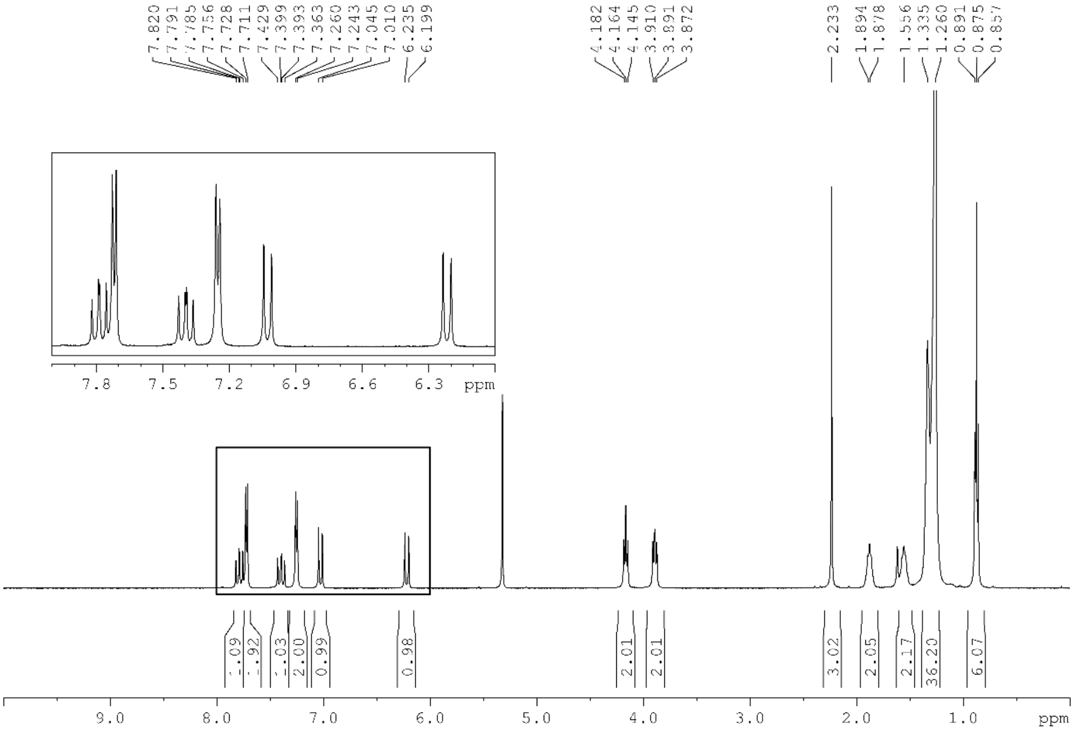

Figure S12. ^{1}H NMR (400 MHz, CD_2Cl_2) spectrum of **B-ref** at 295 K.

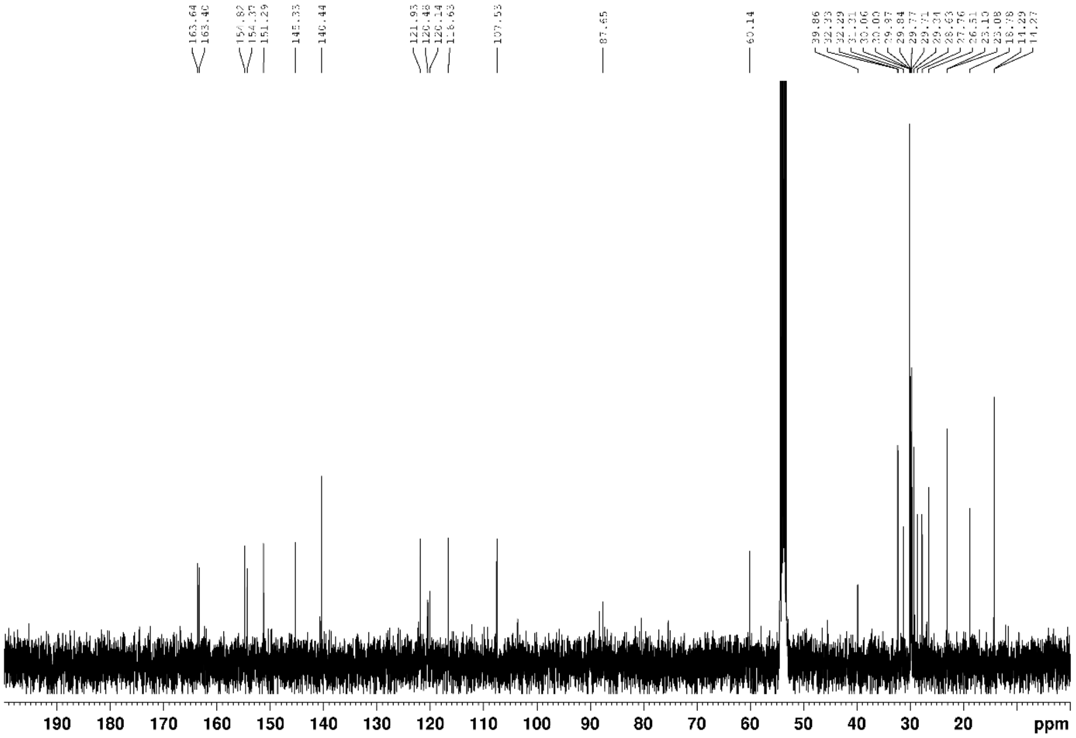

Figure S13. ^{13}C NMR (101 MHz, CD_2Cl_2) spectrum of **B-ref** at 295 K.

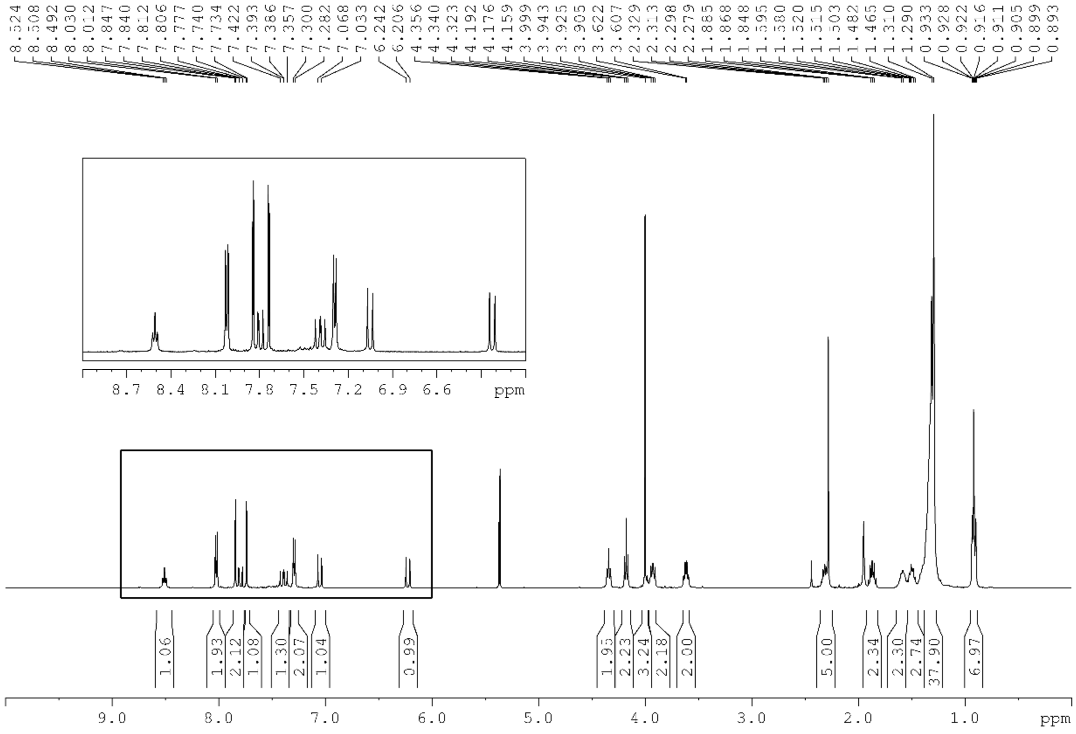

Figure S14. ^{1}H NMR (400 MHz, CD_2Cl_2) spectrum of **Ba** at 295 K.

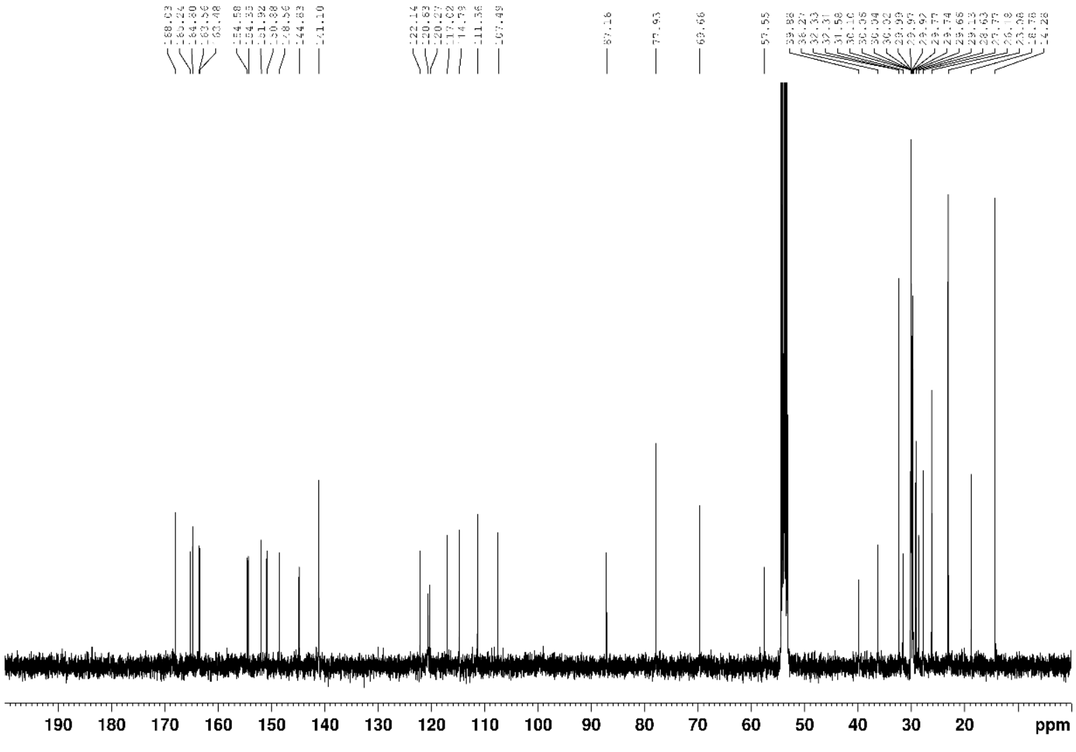

Figure S15. ^{13}C NMR (101 MHz, CD_2Cl_2) spectrum of **Ba** at 295 K.

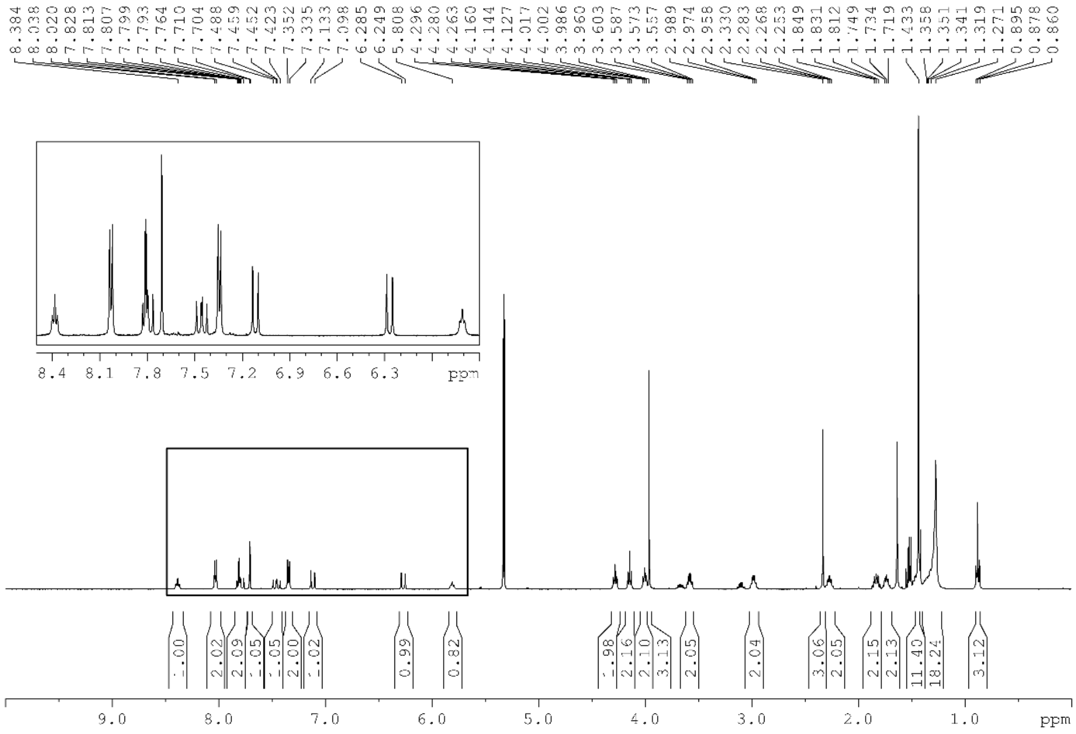

Figure S16. ^{1}H NMR (400 MHz, CD_2Cl_2) spectrum of **Bb** at 295 K.

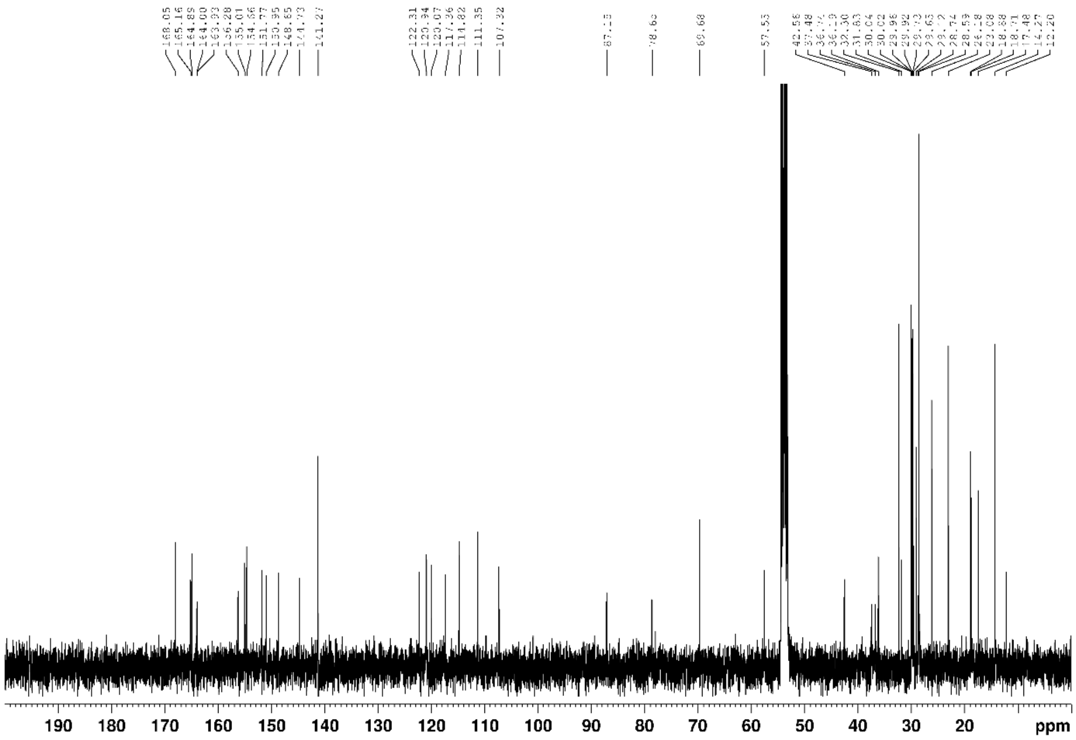

Figure S17. ^{13}C NMR (101 MHz, CD_2Cl_2) spectrum of **Bb** at 295 K.

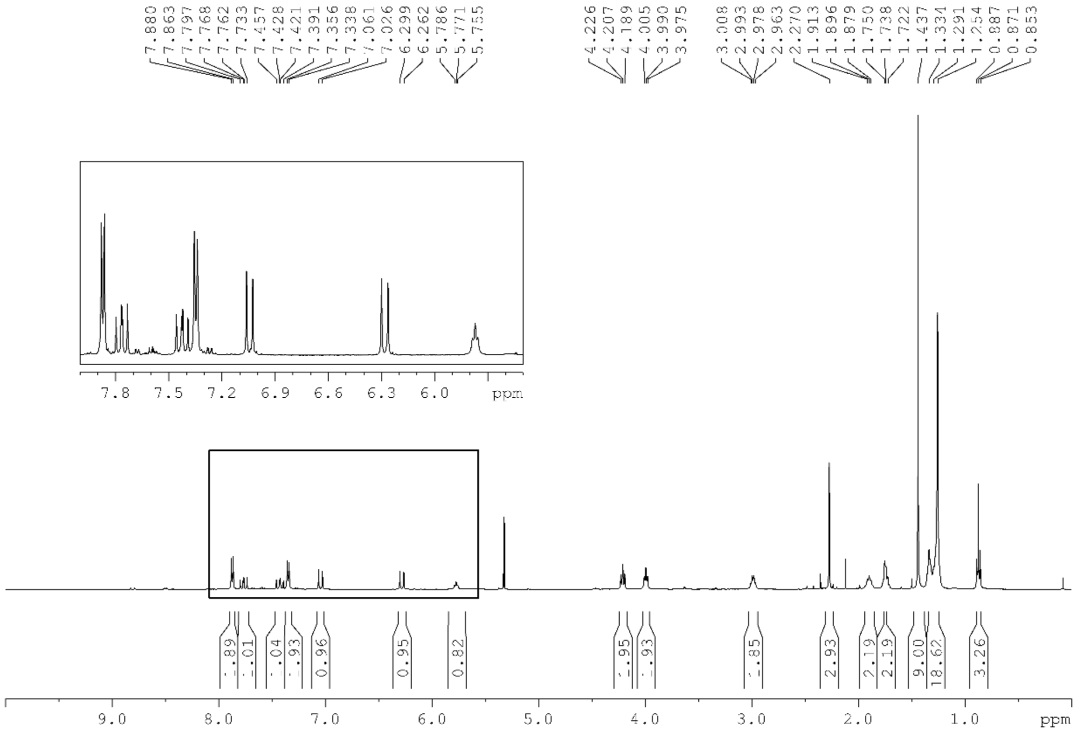

Figure S18. ^{1}H NMR (400 MHz, CD_2Cl_2) spectrum of **Bc** at 295 K.

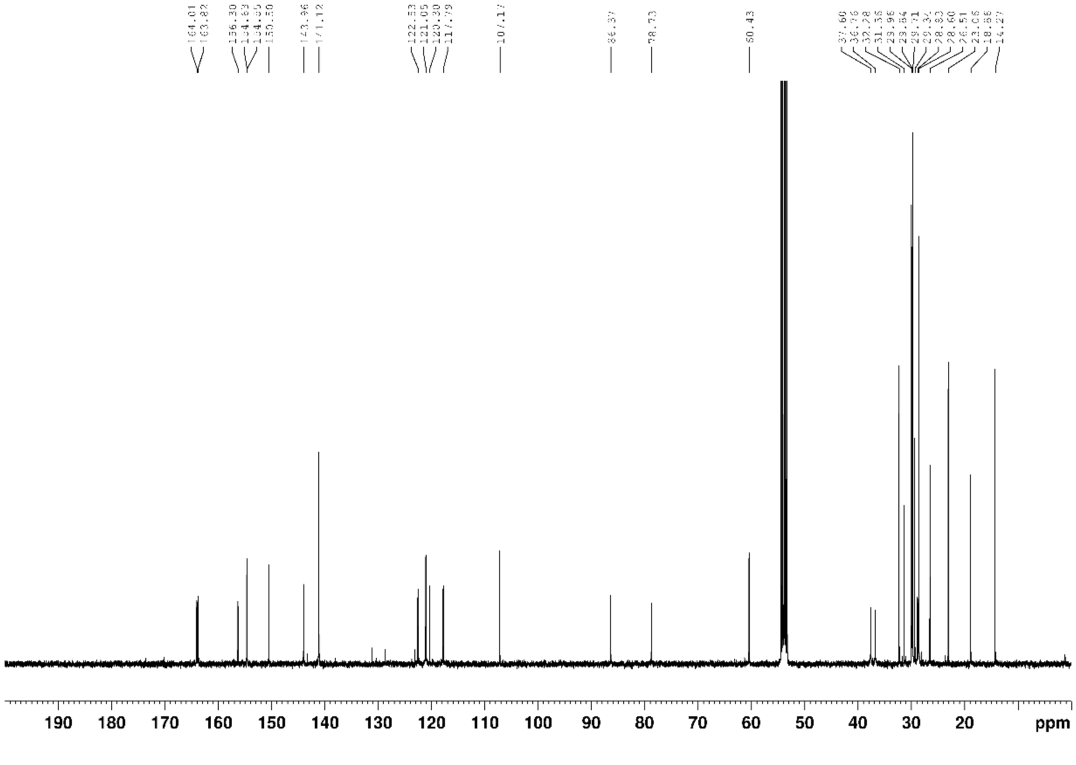

Figure S19. ^{13}C NMR (101 MHz, CD_2Cl_2) spectrum of **Bc** at 295 K.

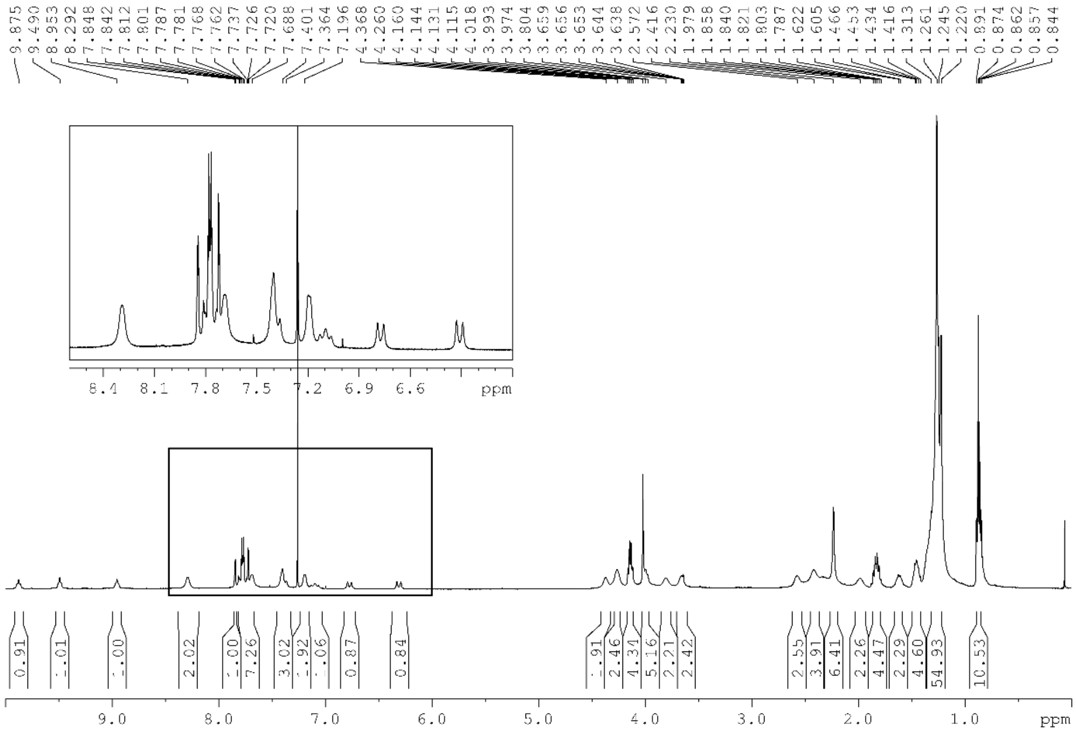

Figure S20. ^1H NMR (400 MHz, $CDCl_3$) spectrum of **RB-OMe** at 295 K.

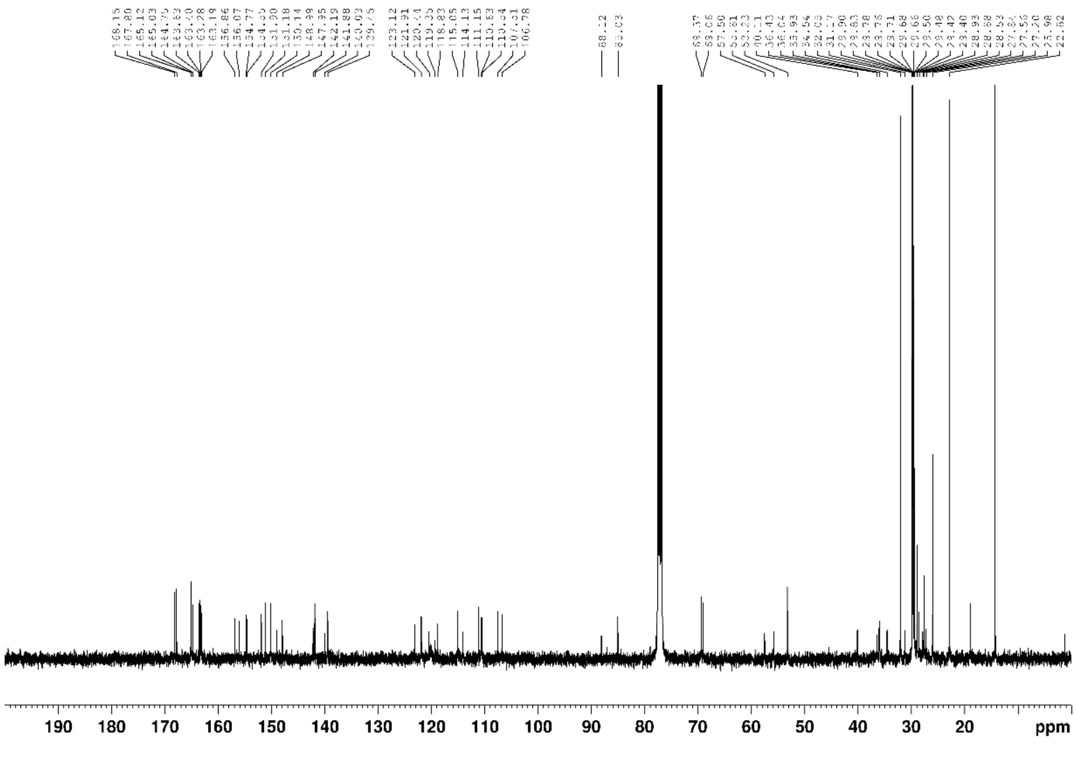

Figure S21. ^{13}C NMR (101 MHz, $CDCl_3$) spectrum of **RB-OMe** at 295 K.

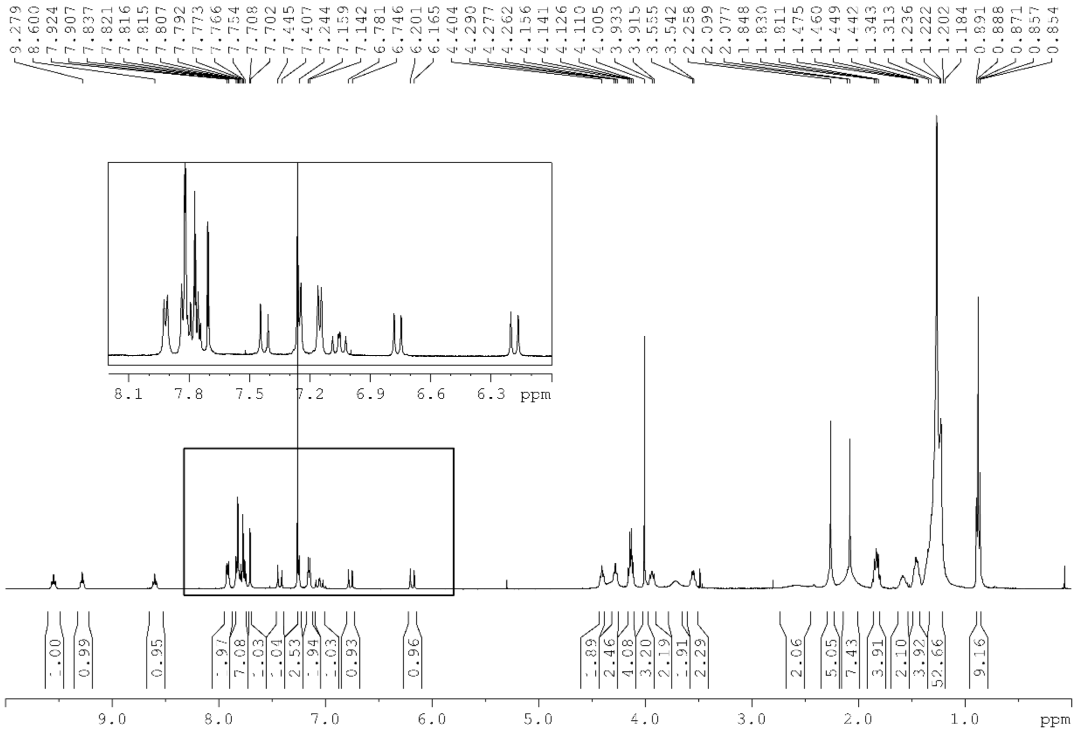

Figure S22. ^{1}H NMR (400 MHz, $CDCl_3$) spectrum of **BR-OMe** at 295 K.

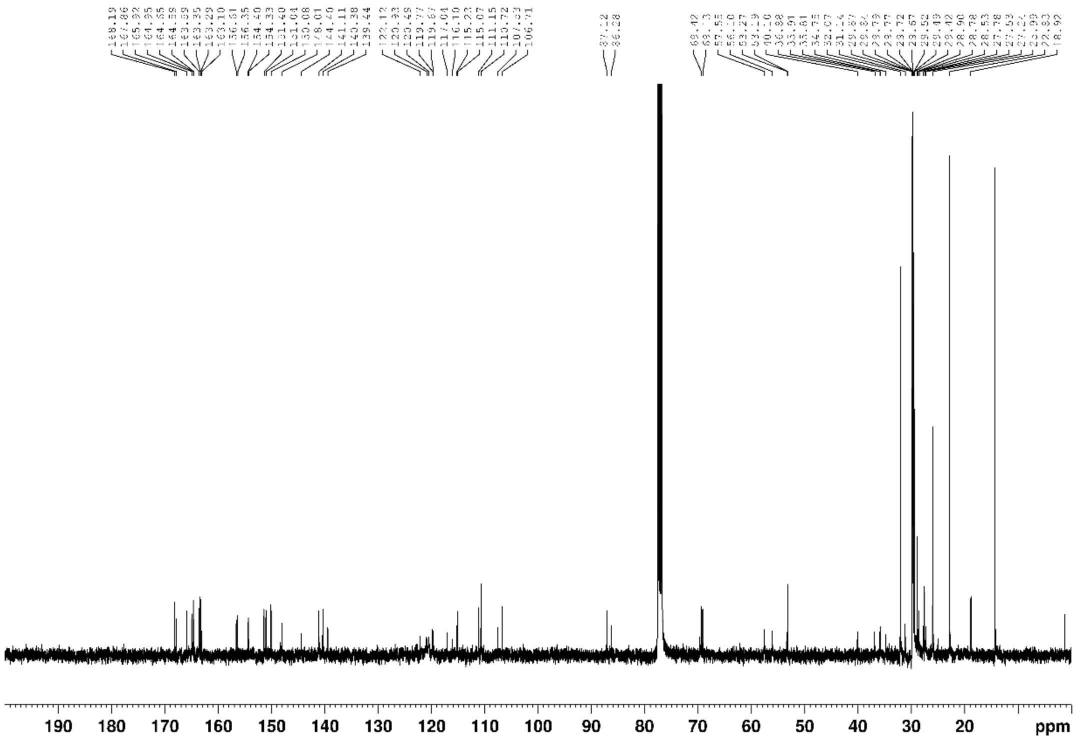

Figure S23. ^{13}C NMR (101 MHz, $CDCl_3$) spectrum of **BR-OMe** at 295 K.

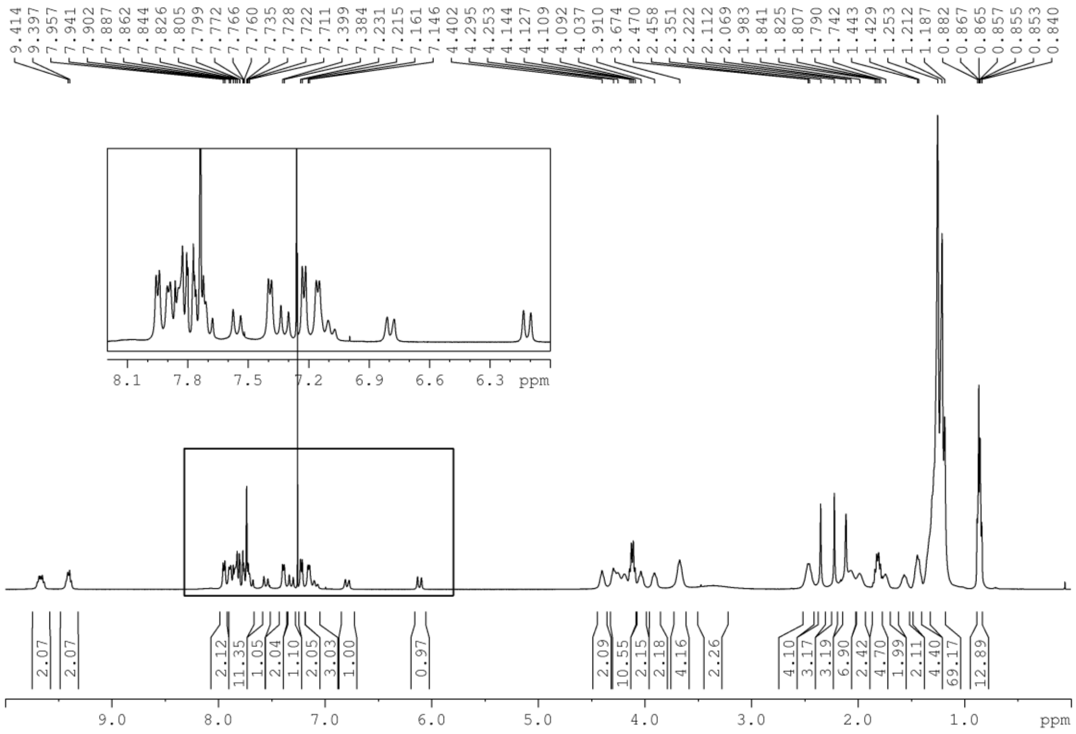

Figure S24. ^{1}H NMR (400 MHz, $CDCl_3$) spectrum of **BRR** at 295 K.

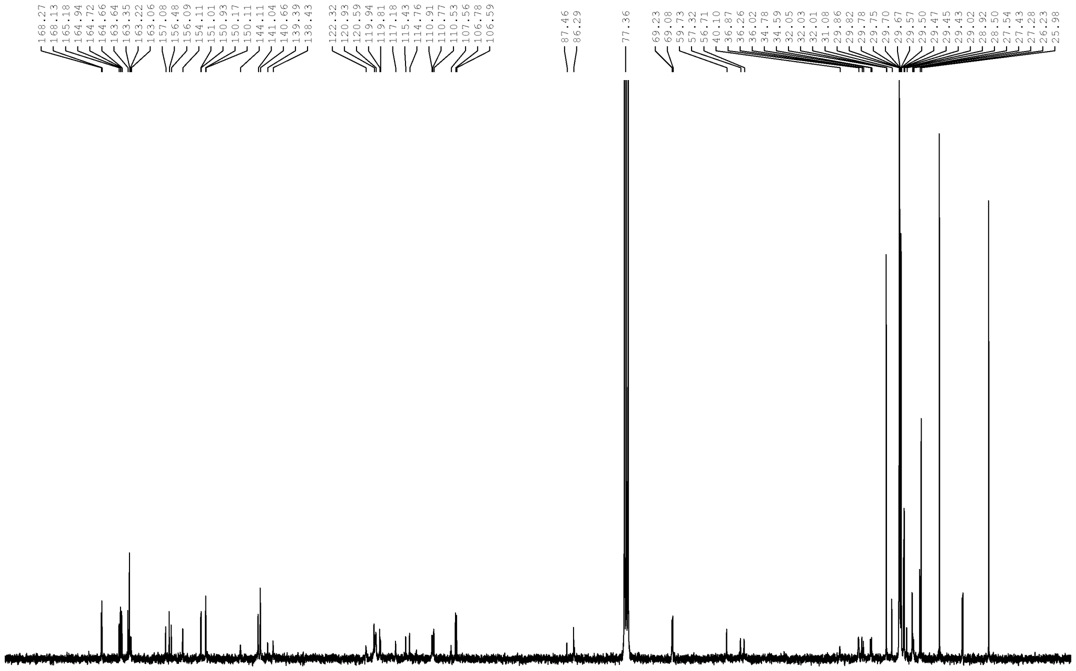

Figure S25. ^{13}C NMR (101 MHz, $CDCl_3$) spectrum of **BRR** at 295 K.

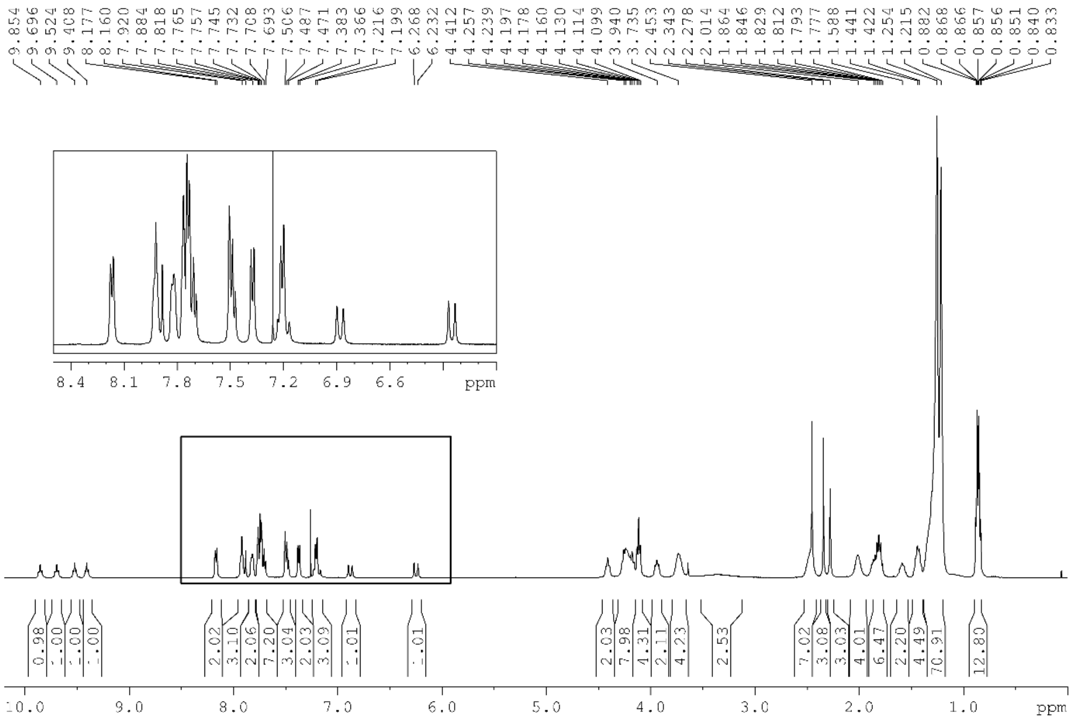

Figure S26. ^{1}H NMR (400 MHz, $CDCl_3$) spectrum of **RBR** at 295 K.

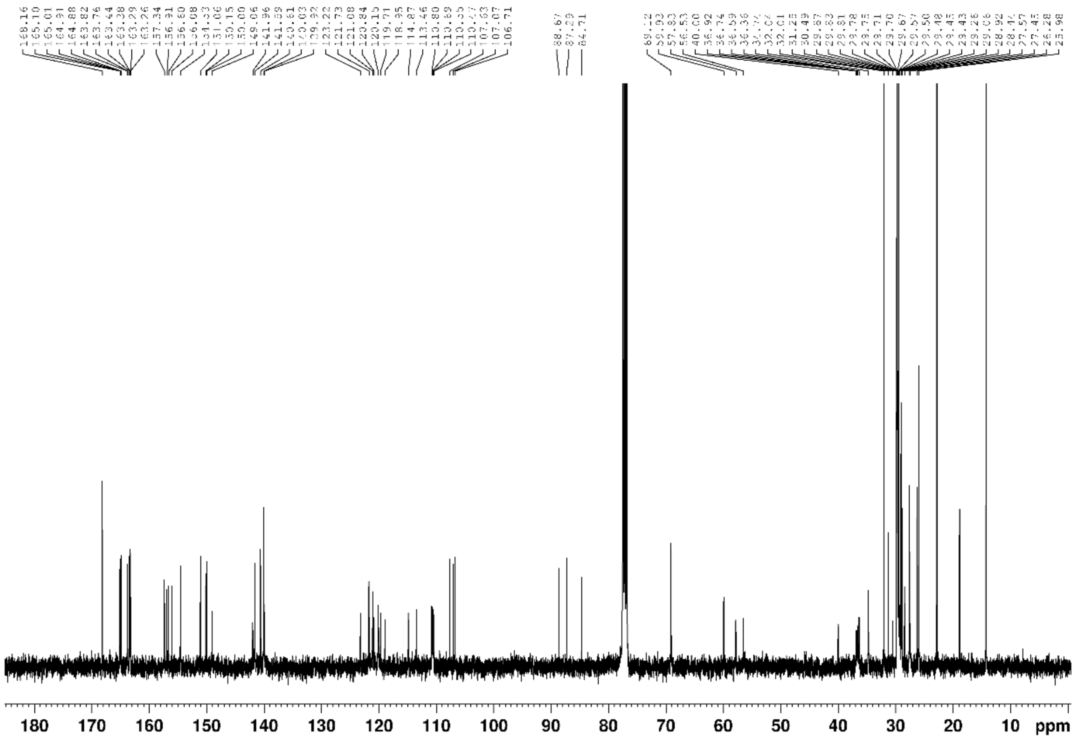

Figure S27. ^{13}C NMR (101 MHz, $CDCl_3$) spectrum of **RBR** at 295 K.

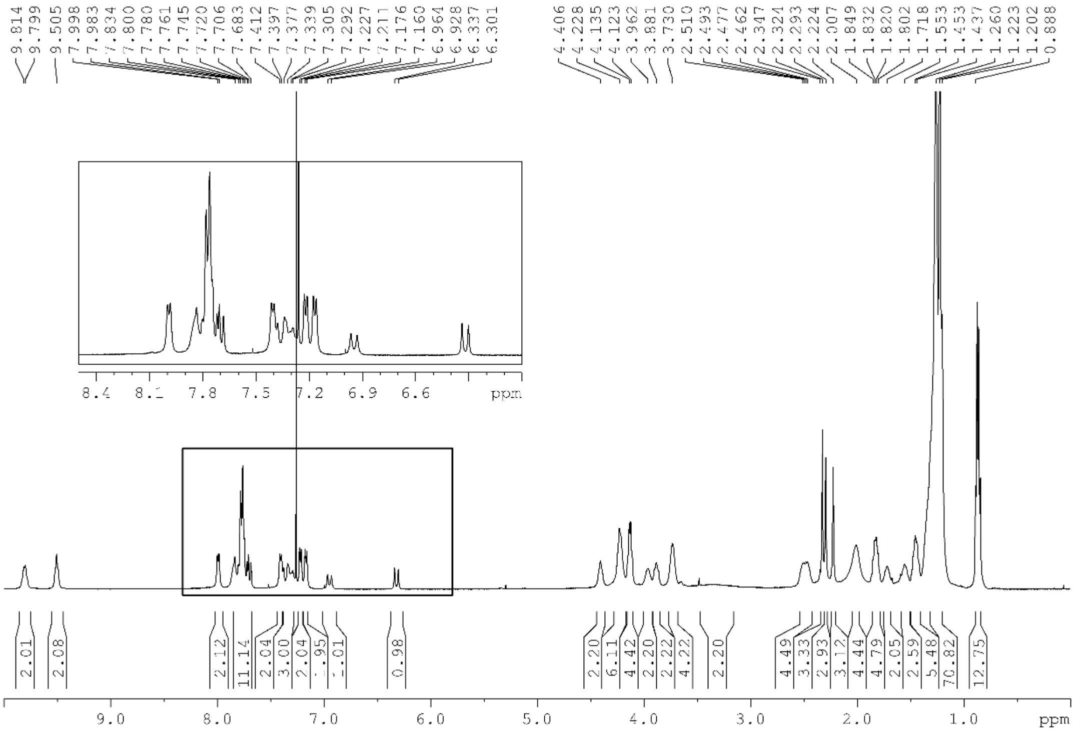

Figure S28. ^1H NMR (400 MHz, $CDCl_3$) spectrum of **RRB** at 295 K.

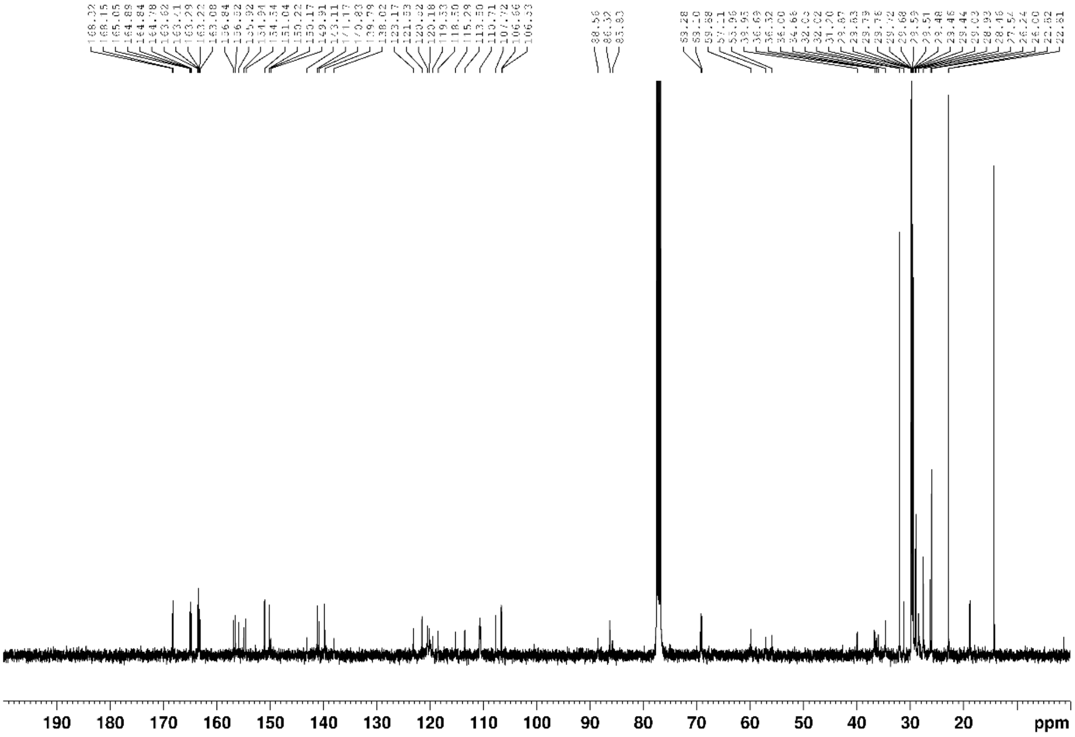

Figure S29. ^{13}C NMR (101 MHz, $CDCl_3$) spectrum of **RRB** at 295 K.

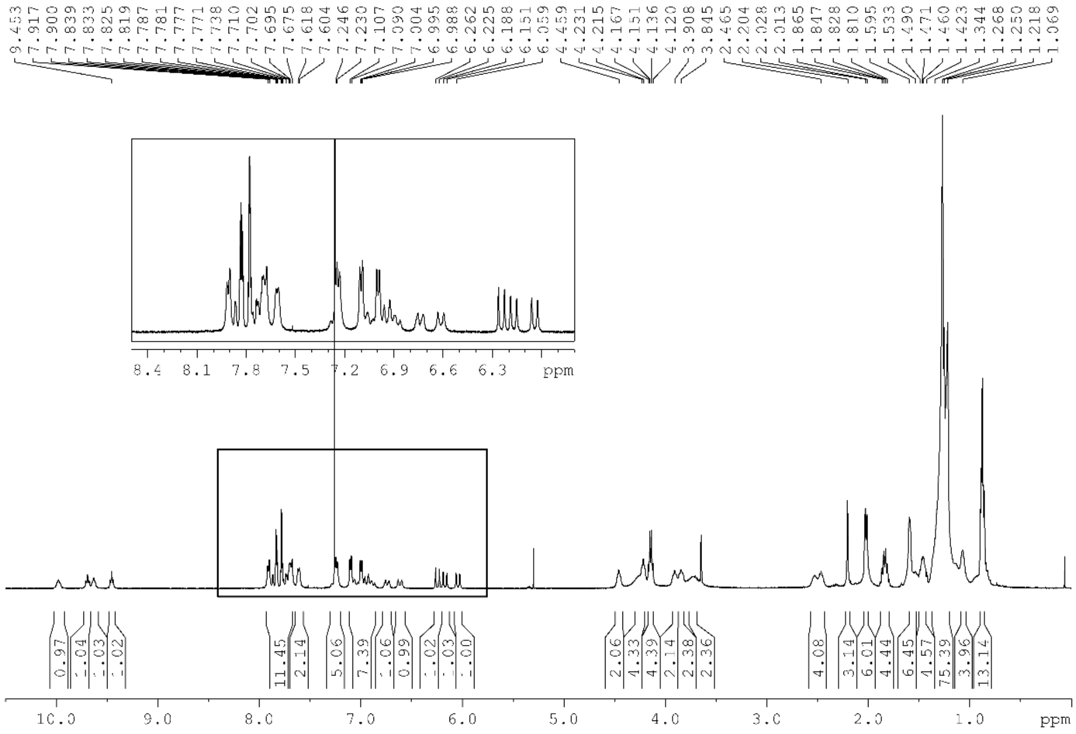

Figure S30. ^{1}H NMR (400 MHz, $CDCl_3$) spectrum of **BBBs** at 295 K.

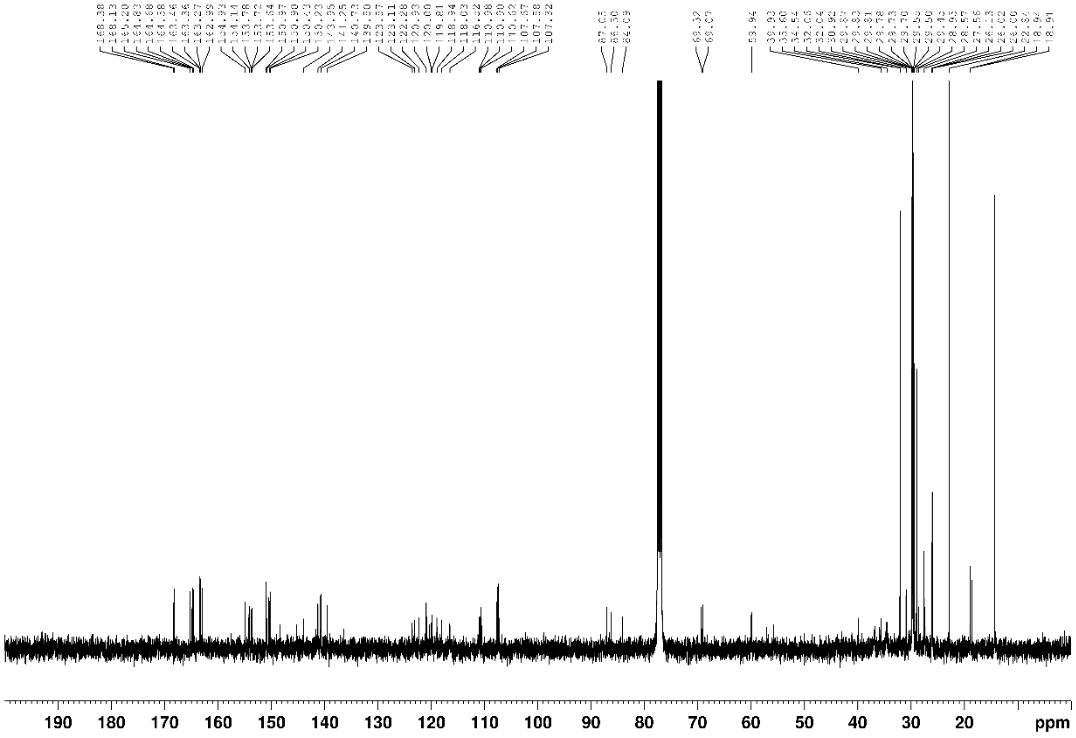

Figure S31. ^{13}C NMR (101 MHz, $CDCl_3$) spectrum of **BBB** at 295 K.

Mass spectra

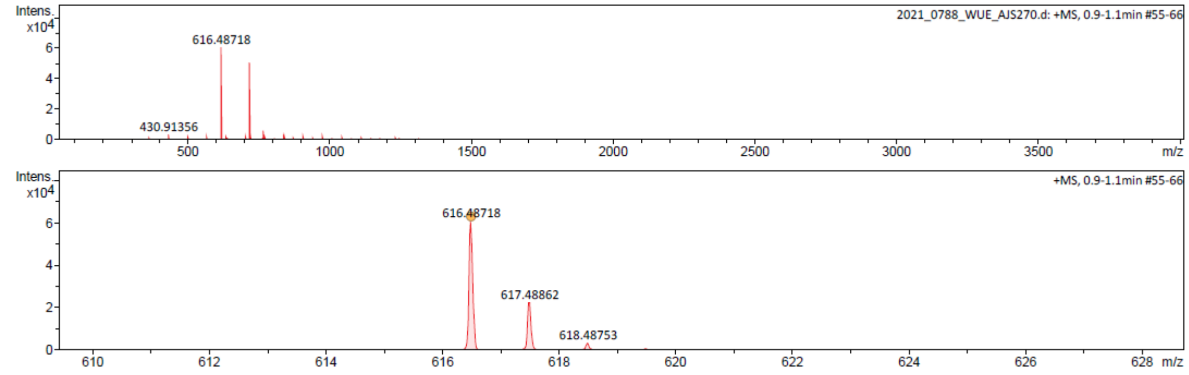

Meas. m/z	#	Ion Formula	m/z	err [ppm]	mSigma	# mSigma	Score	rdb	e⁻ Conf	N-Rule
616.48718	1	C40H62N3O2	616.48420	-4.83	49.0	1	100.00	11.5	even	ok

Figure S32. HRMS (ESI-TOF, MeCN/$CHCl_3$) of **B-ref** [M + H]$^+$.

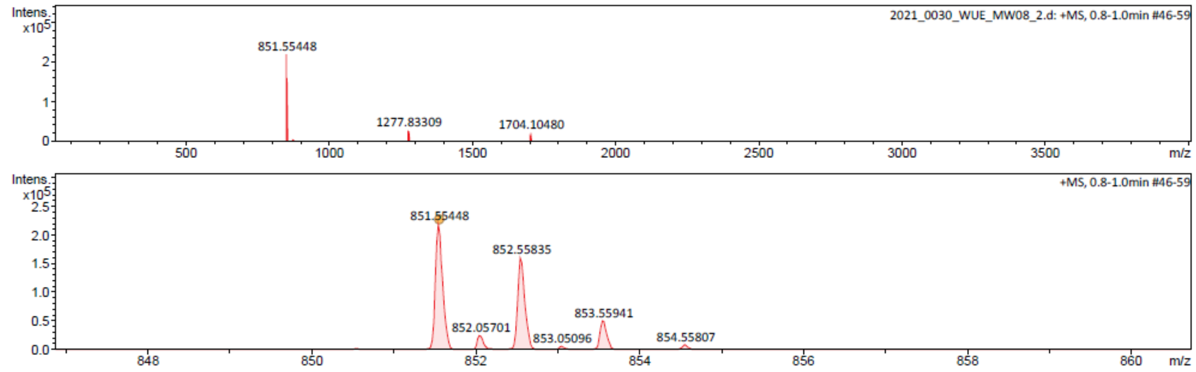

Meas. m/z	#	Ion Formula	m/z	err [ppm]	mSigma	# mSigma	Score	rdb	e⁻ Conf	N-Rule
851.55448	1	C51H73N5O6	851.55554	1.24	80.2	1	100.00	18.0	odd	ok

Figure S33. HRMS (ESI-TOF, MeCN/$CHCl_3$) of **Ba** [M]$^+$.

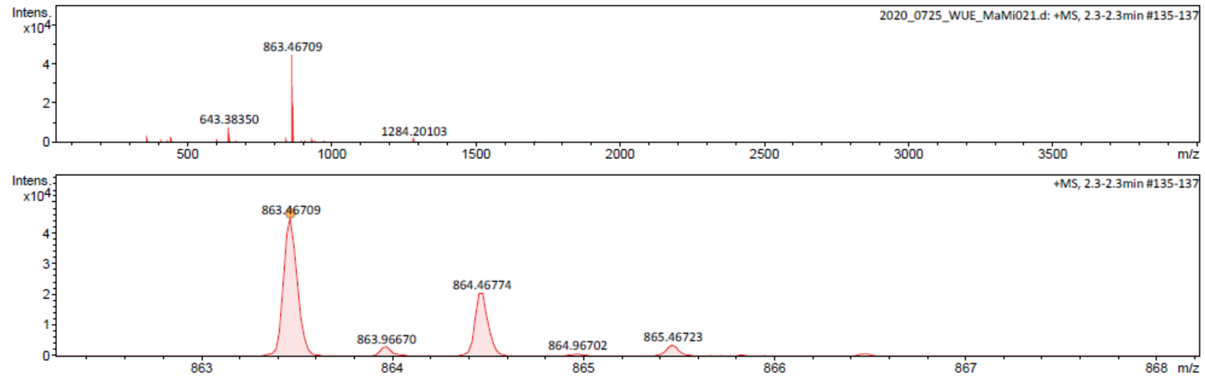

Meas. m/z	#	Ion Formula	m/z	err [ppm]	mSigma	# mSigma	Score	rdb	e⁻ Conf	N-Rule
863.46709	1	C47H64N6NaO8	863.46833	1.44	58.4	1	100.00	18.5	even	ok

Figure S34. HRMS (ESI-TOF, MeCN/$CHCl_3$) of **Bb** [M + Na]$^+$.

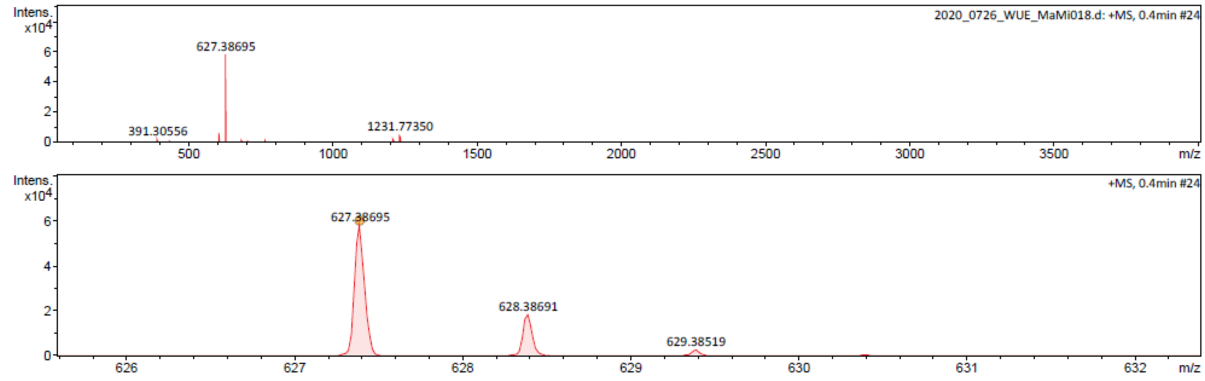

Meas. m/z	#	Ion Formula	m/z	err [ppm]	mSigma	# mSigma	Score	rdb	e⁻ Conf	N-Rule
627.38695	1	C36H52N4NaO4	627.38863	2.67	53.1	1	100.00	12.5	even	ok

Figure S35. HRMS (ESI-TOF, MeCN/$CHCl_3$) of **Bc** [M + Na]$^+$.

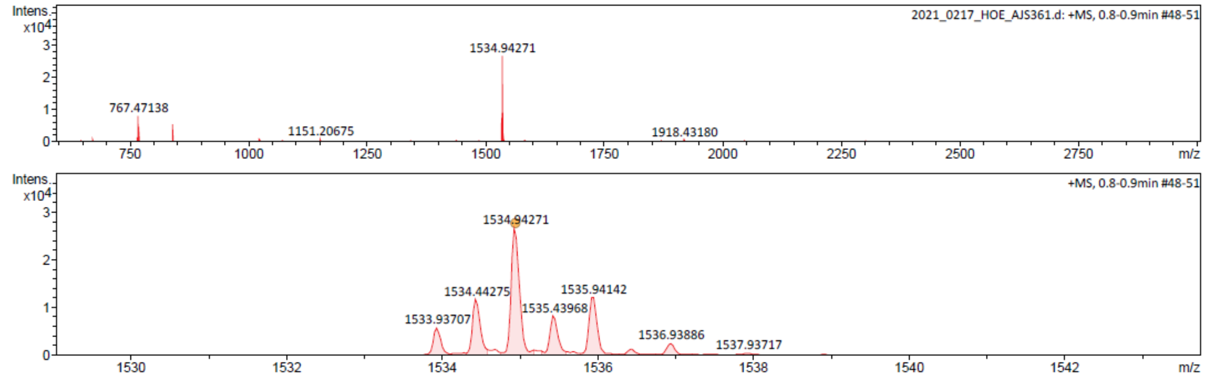

Meas. m/z	#	Ion Formula	m/z	err [ppm]	mSigma	# mSigma	Score	rdb	e⁻ Conf	N-Rule
1534.94271	1	C90H124N11O11	1534.94818	3.57	294.5	1	100.00	34.5	even	ok

Figure S36. HRMS (ESI-TOF, MeCN/$CHCl_3$) of **RB** [M + H]$^+$.

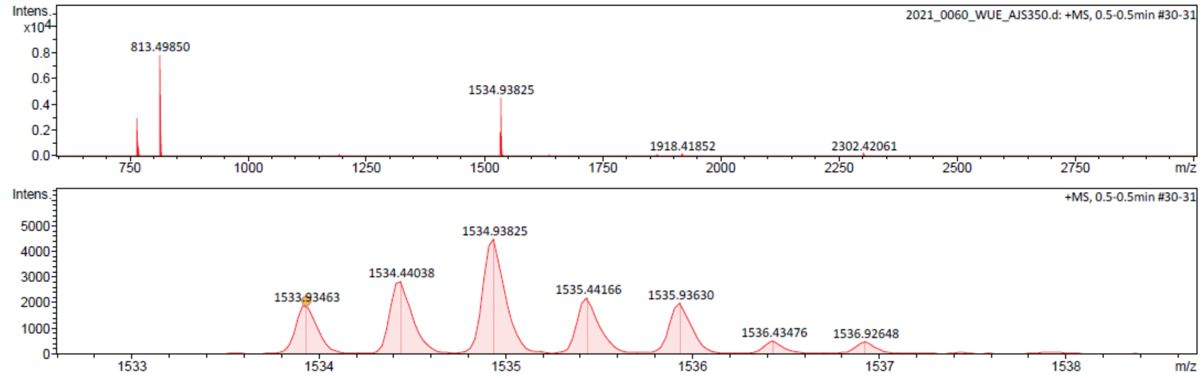

Meas. m/z	#	Ion Formula	m/z	err [ppm]	mSigma	# mSigma	Score	rdb	e⁻ Conf	N-Rule
1533.93463	1	C90H123N11O11	1533.93981	3.38	241.9	1	100.00	35.0	odd	ok

Figure S37. HRMS (ESI-TOF, MeCN/$CHCl_3$) of **BR** [M]$^+$.

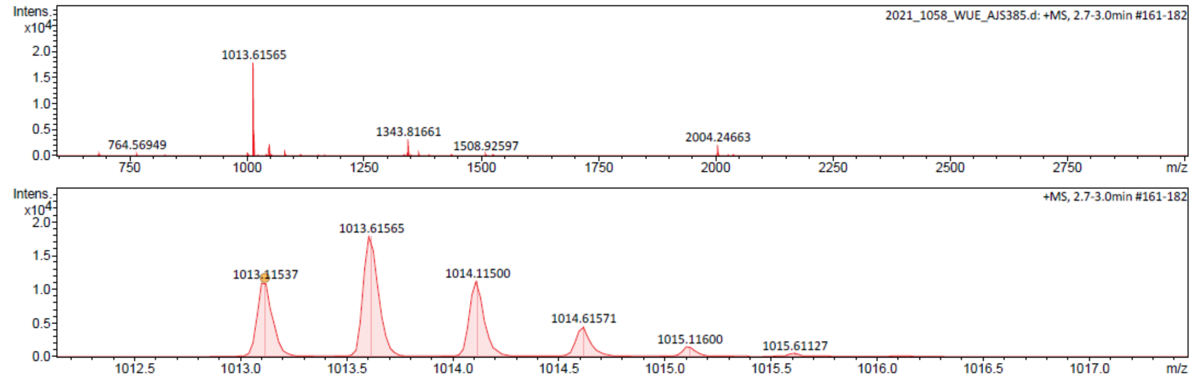

Meas. m/z	#	Ion Formula	m/z	err [ppm]	mSigma	# mSigma	Score	rdb	e⁻ Conf	N-Rule
1013.11537	1	C118H161N15Na2O12	1013.11168	-3.65	66.2	1	100.00	45.0	even	ok

Figure S38. HRMS (ESI-TOF, MeCN/$CHCl_3$) of **RRB** $[M + 2Na]^{2+}$.

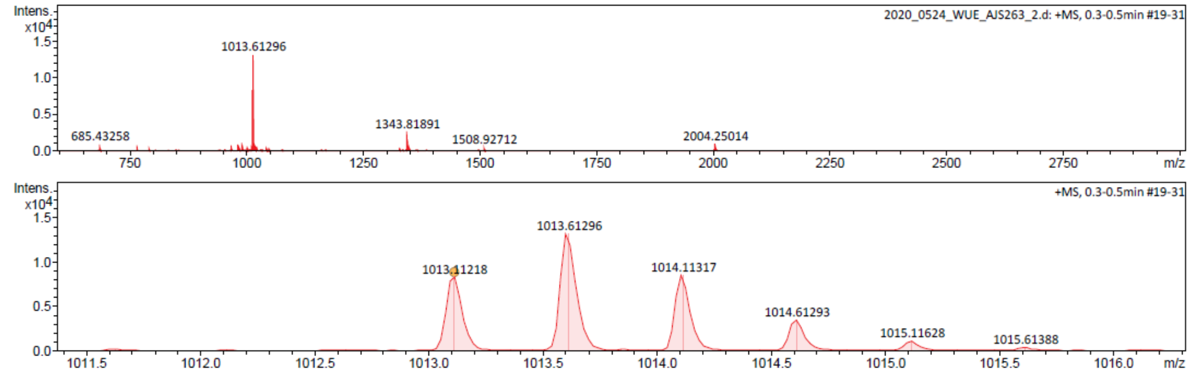

Meas. m/z	#	Ion Formula	m/z	err [ppm]	mSigma	# mSigma	Score	rdb	e⁻ Conf	N-Rule
1013.11218	1	C118H161N15Na2O12	1013.11168	-0.50	53.5	1	100.00	45.0	even	ok

Figure S39. HRMS (ESI-TOF, MeCN/$CHCl_3$) of **RBR** $[M + 2Na]^{2+}$.

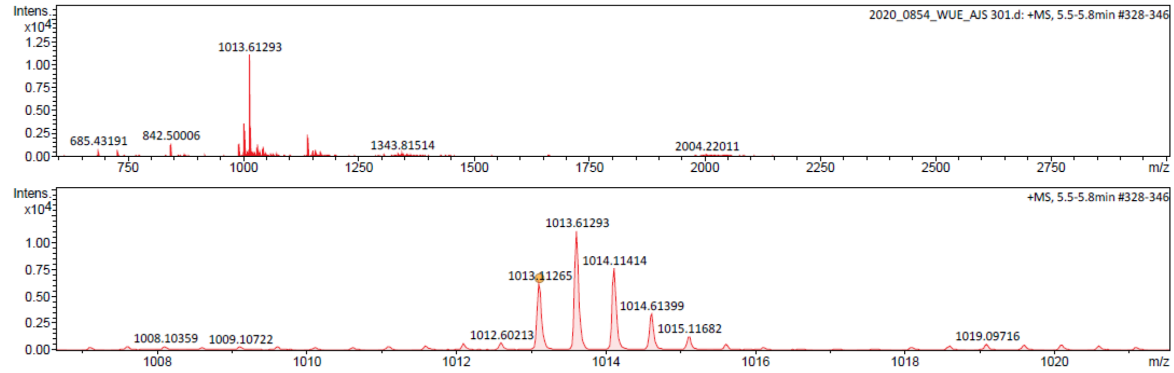

Meas. m/z	#	Ion Formula	m/z	err [ppm]	mSigma	# mSigma	Score	rdb	e⁻ Conf	N-Rule
1013.11265	1	C118H161N15Na2O12	1013.11168	-0.96	73.0	1	100.00	45.0	even	ok

Figure S40. HRMS (ESI-TOF, MeCN/$CHCl_3$) of **RRB** $[M + 2Na]^{2+}$.

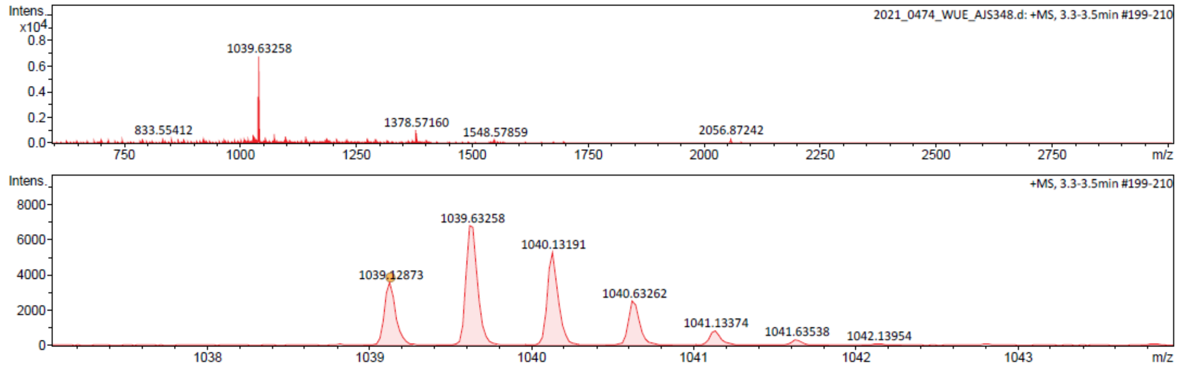

Meas. m/z	#	Ion Formula	m/z	err [ppm]	mSigma	# mSigma	Score	rdb	e¯ Conf	N-Rule
1039.12873	1	C122H165N15Na2O12	1039.12733	-1.35	82.1	1	100.00	47.0	even	ok

Figure S41. HRMS (ESI-TOF, MeCN/$CHCl_3$) of **BBB** $[M+2Na]^{2+}$.

Additional References

S1) X. Hu, A. Schulz, J. O. Lindner, M. Grüne, D. Bialas, F. Würthner, *Chem. Sci.* **2021**, *12*, 8342–8352.
S2) X. Hu, J. O. Lindner, F. Würthner, *J. Am. Chem. Soc*. **2020**, *142*, 3321–3325.
S3) G. R. Fulmer, A. J. M. Miller, N. H. Sherden, H. E. Gottlieb, A. Nudelman, B. M. Stoltz, J. E. Bercaw, K. I. Goldberg, *Organometallics* **2010**, *29*, 2176–2179.
S4) A. D. Becke, J. *Chem. Phys*. **1993**, *98*, 1372–1377.
S5) F. Weigend, R. Ahlrichs, *Phys. Chem. Chem. Phys*. **2005**, *7*, 3297–3305.
S6) Gaussian 09, Revision A.02, M. J. Frisch, G. W. Trucks, H. B. Schlegel, G. E. Scuseria, M. A. Robb, J. R. Cheeseman, G. Scalmani, V. Barone, G. A. Petersson, H. Nakatsuji, X. Li, M. Caricato, A. Marenich, J. Bloino, B. G. Janesko, R. Gomperts, B. Mennucci, H. P. Hratchian, J. V. Ortiz, A. F. Izmaylov, J. L. Sonnenberg, D. Williams-Young, F. Ding, F. Lipparini, F. Egidi, J. Goings, B. Peng, A. Petrone, T. Henderson, D. Ranasinghe, V. G. Zakrzewski, J. Gao, N. Rega, G. Zheng, W. Liang, M. Hada, M. Ehara, K. Toyota, R. Fukuda, J. Hasegawa, M. Ishida, T. Nakajima, Y. Honda, O. Kitao, H. Nakai, T. Vreven, K. Throssell, J. A. Montgomery, Jr., J. E. Peralta, F. Ogliaro, M. Bearpark, J. J. Heyd, E. Brothers, K. N. Kudin, V. N. Staroverov, T. Keith, R. Kobayashi, J. Normand, K. Raghavachari, A. Rendell, J. C. Burant, S. S. Iyengar, J. Tomasi, M. Cossi, J. M. Millam, M. Klene, C. Adamo, R. Cammi, J. W. Ochterski, R. L. Martin, K. Morokuma, O. Farkas, J. B. Foresman, and D. J. Fox, Gaussian, Inc., Wallingford CT, 2016.
S7) J.-D. Chai, M. Head-Gordon, *J. Chem. Phys*. **2008**, *128*, 084106.
S8) S. Grimme, J. Antony, S. Ehrlich, H. Krieg, *J. Chem. Phys*. **2010**, *132*, 154104.
S9) G. Seybold, G. Wagenblast, *Dyes Pigm*. **1989**, *11*, 303–317.
S10) A. Alessi, M. Salvalaggio, G. Ruzzon, *J. Lumin*. **2013**, *134*, 385–389.
S11) K. A. Kistler, F. C. Spano, S. Matsika, *J Phys Chem B* **2013**, *117*, 2032-2044.
S12) T. Lu, F. Chen, *J. Comput. Chem.* **2012**, *33*, 580-592.
